# Genome-wide DNA methylation analysis of pulmonary function in middle and old-aged Chinese monozygotic twins

**DOI:** 10.1186/s12931-021-01896-5

**Published:** 2021-11-22

**Authors:** Tong Wang, Weijing Wang, Weilong Li, Haiping Duan, Chunsheng Xu, Xiaocao Tian, Dongfeng Zhang

**Affiliations:** 1 Department of Epidemiology and Health Statistics, the College of Public Health of Qingdao University, NO. 308 Ning Xia Street, Qingdao, 266071 Shandong Province People’s Republic of China; 2 Population Research Unit, Faculty of Social Sciences, University of Helsinki, Helsinki, Finland; 3 Qingdao Municipal Center for Disease Control and Prevention, Qingdao, Shandong Province People’s Republic of China; 4 Qingdao Institute of Preventive Medicine, Qingdao, Shandong Province People’s Republic of China

**Keywords:** DNA methylation, Epigenetics, Monozygotic twins, Pulmonary function

## Abstract

**Background:**

Previous studies have determined the epigenetic association between DNA methylation and pulmonary function among various ethnics, whereas this association is largely unknown in Chinese adults. Thus, we aimed to explore epigenetic relationships between genome-wide DNA methylation levels and pulmonary function among middle-aged Chinese monozygotic twins.

**Methods:**

The monozygotic twin sample was drawn from the Qingdao Twin Registry. Pulmonary function was measured by three parameters including forced expiratory volume the first second (FEV1), forced vital capacity (FVC), and FEV1/FVC ratio. Linear mixed effect model was used to regress the methylation level of CpG sites on pulmonary function. After that, we applied Genomic Regions Enrichment of Annotations Tool (GREAT) to predict the genomic regions enrichment, and used comb-p python library to detect differentially methylated regions (DMRs). Gene expression analysis was conducted to validate the results of differentially methylated analyses.

**Results:**

We identified 112 CpG sites with the level of *P* < 1 × 10^–4^ which were annotated to 40 genes. We identified 12 common enriched pathways of three pulmonary function parameters. We detected 39 DMRs located at 23 genes, of which *PRDM1* was related to decreased pulmonary function, and *MPL*, *LTB4R2*, and *EPHB3* were related to increased pulmonary function. The gene expression analyses validated *DIP2C*, *ASB2*, *SLC6A5*, and *GAS6* related to decreased pulmonary function.

**Conclusion:**

Our DNA methylation sequencing analysis on identical twins provides new references for the epigenetic regulation on pulmonary function. Several CpG sites, genes, biological pathways and DMRs are considered as possible crucial to pulmonary function.

**Supplementary Information:**

The online version contains supplementary material available at 10.1186/s12931-021-01896-5.

## Introduction

Pulmonary function is determined as an important predictor of cardiovascular health [1] and mortality [2], which declines with increasing age after the third decade of lifetime [3]. Accelerated decline in pulmonary function has immense impact on individual and social economy [4]. Pulmonary function can be influenced by a variety of factors. Traditional epidemiologic studies have widely investigated the relationship of environmental factors, such as cigarette smoking [5] and air pollution [6] with pulmonary function. Besides, family-based study [7] and genome-wide association study (GWAS) [8] have estimated the heritability of pulmonary function ranging from 0.42 to 0.71, indicating genetic contribution to the variation of pulmonary function.

Currently, an increasing number of GWASs have smoothed the way for discovering human genetic variants linked to pulmonary function which are quantified by forced expiratory volume in one second (FEV1), forced vital capacity (FVC), and FEV1/FVC ratio [9]. Yet the reported nucleotide-level polymorphisms could explain a limited proportion of pulmonary function variation [10] (5.0% for FEV1, 3.4% for FVC, and 9.2% for FEV1/FVC) compared with the estimated heritability, suggesting that other gene-regulatory mechanisms such as epigenetics might also be at play. Epigenetics is the study of heritable phenotype alterations that do not involve changes in the DNA sequence [11], and the epigenetic changes include DNA methylation, histone modification and non-coding RNA. Previous epigenome-wide association studies (EWASs) have investigated the association between DNA methylation and pulmonary function among various ethnic population but only a limited amount of significant genomic sites have been revealed [4, 12–14]. Besides, expect one study based on monozygotic (MZ) twin design, most of previous studies were conducted based on general population, which could not control the genetic effect and early life milieu including intrauterine environment on epigenetic changes [15].

As the genetic makeup is perfectly matched within pair, the monozygotic twins serve as optimal and valuable samples for EWAS on complex diseases and phenotypes [16]. The genetic influences on epigenetic changes are cancelled out in the discordant MZ twins design, thus the differential DNA methylation triggered by environmental factors could be identified [17]. The Chinese population is different from the other ethnics of the world in terms of genetic background, environmental exposure and lifestyle. However, there is no EWAS of pulmonary function in the Chinese twins published present. Thereby, we performed an EWAS to identify the association between DNA methylation variants and pulmonary function among Chinese monozygotic twin pairs.

## Materials and methods

### Samples and study procedures

The discordant identical twin pairs are sub-sample of twins derived from Qingdao Twin Registry [18] conducted by Qingdao Centers for Disease Control and Prevention. The details of sample recruitment have been described elsewhere [19]. A total of 68 twin pairs which were conducted DNA methylation sequencing using the reduced representation bisulfite sequencing (RRBS) were included in the sample. After excluded twin pairs with incomplete measurement of pulmonary function (n = 1) and participants with minimal absolute values of intra-pair difference in pulmonary function score (ΔFEV1 < 0.1, n = 7; ΔFVC < 0.1, n = 8, and ΔFEV1/FVC < 0.05, n = 23), complete monozygotic twin pairs who met the criteria were included in the study, including 60 twin pairs for FEV1(34 male and 26 female pairs), 59 twin pairs for FVC (34 male and 25 female pairs), and 44 twin pairs for FEV1/FVC (21 male and 23 female pairs). Informed written consents were obtained from all participants. Regional Ethics Committee of the Qingdao Centers for Disease Control and Prevention Institutional Review Boards has approved this study.

Pulmonary function including FEV1 and FVC (liters) was assessed by the electronic hand-held spirometer (Micro 0102). Trained investigators calibrated the spirometer before measurement every morning. Based on the standard procedure of spirometry, each participant performed two maneuvers in standing state twice, and best trial data were applied to further analysis. The ratio FEV1/FVC was calculated according to the above measurements.

### DNA methylation analysis

The Cetyltrimethyl Ammonium Bromide was used to extract genomic DNA from whole blood. DNA methylation library was constructed using RRBS by Biomarker Technologies Corporation, Beijing, China (http://www.biomarker.com.cn/). Firstly, genomic DNA was digested with Mspl restriction enzyme. After digesting, the 5′ CG overhangs were repaired, and A-tails were added. Then the DNA was loaded on an agarose gel, and 230–380 bp long (including 100 bp adaptor) fragments were sort out for next bisulfite conversion using NEXTflex Bisulfite-Seq Kit (Bioo Scientific, Austin, TX, USA). After all, the bisulfite converted DNA was amplified with PCR. The reduced representation bisulfite sequencing was conducted using Illumina HiSeq X Ten (Illumina Inc., San Diego, CA, USA).

### Data preprocessing

Our previous study has detailed the data preprocessing [20, 21]. In brief, the raw data were first trimmed and mapped to Genome Reference Consortium Human Build 37 (hg19) by Bismark [22]. The mapping output from Bismark was then imported to BiSeq (R package) [23] to detect the methylation level. To reduce bias, the coverage was restricted to 90% quantile, and CpG sites with beyond ten missing observations or average methylation beta value < 0.01 were removed. We used logit transformation to transform the beta value to M-value for conducting further differential methylation analyses.

### Cell-type composition

Because the DAN sample extracted from the whole blood including distinct cell types which might result in false discoveries. We applied ReFACTor [24] method to control the cellular heterogeneity impact on DNA methylation. ReFACTor is based on principal component analysis and calculates the linear transformations of cell-type composition as principal component analysis components. We selected the top five components as covariates to control cell-type heterogeneity for the subsequent analyses.

### Statistical analysis

#### Epigenome-wide association analyses

For single CpG analysis, linear mixed effect models were applied to regress methylation level on pulmonary function adjusting for cell-type composition and other confounding factors (FEV1: diastolic pressure; FVC: none; FEV1/FVC: diastolic and systolic pressure) as fixed effects and twin pairing variable as a random effect, based on the co-twin design as proposed by Tan et al. [16]. The smoking status of in-pair twins were almost consistent in sample. The number of smoking twins was 22 for FEV1 and FVC and 15 for FEV1/FVC, the number of non-smoking twins was 32 for FEV1, 31 for FVC, and 25 for FEV1/FVC, and the number of inconsistent smoking status twins was 6 for FEV1 and FVC, 4 for FEV1/FVC. We added the smoking status as fixed effects to control it. False discovery rate (FDR) [25] was calculated to solve multiplicity problem. We defined the significance of genome-wide as FDR < 0.05, and conducted these analyses by R software (version 4.1.0).

#### Genomic regions enrichment analysis

Genomic regions enrichment analysis was performed using Genomic Regions Enrichment of Annotations Tool (GREAT) to examine the enrichment of identified methylation sites (*P* < 0.05) in the functional significance of cis-regulatory regions [26]. GREAT is able to properly incorporate distal binding sites and control for false positives using a binomial test over the input genomic regions. Annotation of GREAT is based on Genome Reference Consortium Human Build 37 (hg19).

#### Detecting differentially methylated regions (DMRs)

Based on bisulfite-sequencing data with *P*-values from EWAS result, the significant differentially methylated regions (DMRs) for pulmonary function were identified using *comb-p* python library proposed by Petersen et al. [27]. This method first combined adjacent *P*-values as weighted according to the calculated auto-correlation, then performed Benjamini–Hochberg false discovery adjustment to find regions of significant enrichment. The documentation and implementation of *comb-p* python library are available at website [28] https://github.com/brentp/combined-pvalues. The analyses of DMRs were conducted by Python software (version 3.8.8).

#### Gene expression analyses

##### Weighted gene co-expression network analyses (WGCNA)

We used R software (version 4.1.0) to perform weighted correlation network analysis such as co-expression network analysis of gene expression data through WGCNA package [29–31]. In brief, we firstly constructed a gene co-expression network, and then used dynamic tree cut to identify modules. Next, we related modules to pulmonary function indices. Finally, we used DAVID [32, 33] tool to conduct the enrichment analysis of genes clustered in specific modules. The significant enriched terms were defined as a modified fisher exact *P*-value < 0.05.

##### Correlational analysis

We applied Spearman's rank correlation analyses by R software (version 4.1.0) to evaluate the correlation between the gene expression levels of genes where the top CpG sites and DMRs annotated and pulmonary function indices. Statistically significant was defied as *P*-value < 0.05.

## Results

Descriptive statistics of basic characteristics are shown in Additional file 1: Table S1. The number of monozygotic twin pairs involved in our study was 60 for FEV1(34 male pairs), 59 for FVC (34 male pairs), and 44 for FEV1/FVC ratio (21 male pairs). The median age of participants was above 50 years old. The mean (standard deviation, SD) of pulmonary function was 1.98 (0.72) for FEV1, 2.33(0.83) for FVC, and 0.86(0.14) for FEV1/FVC. Most clinical indicators had considerably significant correlation, indicating that our discordant MZ twin design could benefit. And the insignificant intra-pair confounders would be added as covariates in our subsequent association analyses. We drew scatter plots with regression line to illustrate the relationship between intra-differences of pulmonary function (ΔFEV1, ΔFVC, ΔFEV1/FVC) and intra-differences of methylated values of top significant CpG sites (*P* value < 10^−4^, Δ methylated values of CpG sites at corresponding location) in MZ twin pairs (Additional file 2: Table S2, Additional file 3: Fig. S1, Additional file 4: Fig. S2, and Additional file 5: Fig. S3). The Δ methylation value of four CpG sites (f, h, i, j) were positively correlated with ΔFEV1, and the Δ methylation value of seven CpG sites (a, b, c, d, e, g, k) were negatively correlated with ΔFEV1. The Δ methylation value of eleven CpG sites (a, b, c, g, h, i, j, k, m, o, q) were positively correlated with ΔFVC, and the Δ methylation value of six CpG sites (d,e,f,l,n,p) were negatively correlated with ΔFVC. The Δ methylation value of two CpG sites (c,i) were positively correlated with ΔFEV1/FVC ratio, and the Δ methylation value of ten CpG sites (a,b,d,e,f,g,h,j,k,l) were negatively correlated with ΔFEV1/FVC ratio.

### Epigenome-wide association analysis

**Fig. 1:**
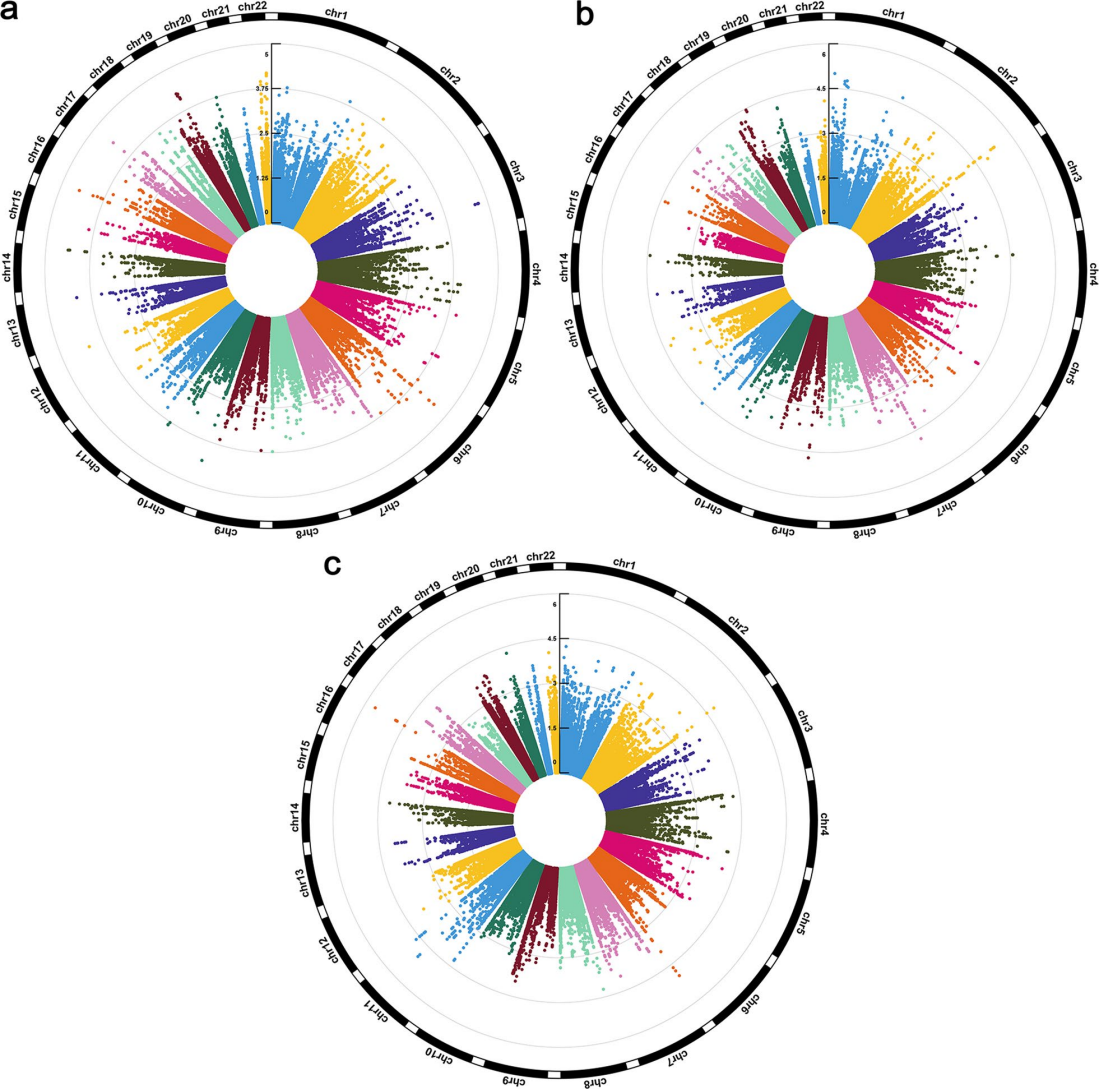
Circular Manhattan plots of FEV1 (**a**), FVC (**b**), and FEV1/FVC (**c**) for single CpG-based epigenome-wide association study. 25 CpGs for FEV1, 56 CpGs for FVC, and 31 CpGs for FEV1/FVC were found as genome-wide significant

The results of EWAS for pulmonary function are shown in Table 1. In analysis of pulmonary function, 25 CpG sites with *P* value < 10^−4^ were identified for FEV1, and the top 25 CpG sites were located at 8 genes, among which 4 (50%) genes *WDR90*, *DIP2C*, *PANX2*, *NUBP2* were associated with pulmonary function. For intra-pair difference in FVC, 56 CpG sites with a *P* value < 10^−4^ were found with 4 sites reaching a *P* value < 10^−5^. And the top CpG sites were located at 21 genes, among which 8 (38%) genes *AP5B1*, *CYP26B1*, *GAS6*, *IL11*, *IRS1*, *IRS2*, *MAD1L1*, *NUAK1* were associated with pulmonary function. Intra-pair methylation difference of FEV1/FVC ratio identified 31 CpG sites with *P* value < 10^−4^. The CpG sites located at 11 genes and the most significant site was located at FENDRR and ENSG00000268388 (chr16: 86,528,639 bp, cor = − 1.93, *P* = 2.27 × 10^−6^). The Manhattan plots of pulmonary function for the *P*-values of each CpG site against its chromosomal location are illustrated in Fig. 1.

**Table 1:** The results of epigenome-wide association study in pulmonary function (*P*-value < 1 × 10^–4^)

Chromosome	Position(bp)	Coefficient	*P*-value	Ensemble gene ID	HGNC symbol
FEV1					
chr3	138,639,540	− 1.93791	1.81E−05		
chr3	138,639,552	− 1.94154	1.87E−05		
chr3	138,639,544	− 1.9364	1.9E−05		
chr3	138,639,520	− 1.92289	2.26E−05		
chr6	43,394,632	− 1.49082	2.87E−05		
chr6	43,394,620	− 1.48934	2.9E−05		
chr16	706,133	0.287479	3.29E−05	ENSG00000161996	*WDR90*
chr14	104,008,425	− 1.59238	4.23E−05		
chr6	43,394,652	− 1.47384	4.29E−05		
chr6	43,394,599	− 1.56562	4.39E−05		
chr17	40,997,066	− 0.79359	4.44E−05	ENSG00000131480	*AOC2*
chr14	104,008,420	− 1.19154	4.65E−05		
chr10	527,775	− 1.12976	4.89E−05	ENSG00000151240	*DIP2C*
chr19^*^	45,721,153	− 0.40397	5.27E−05	ENSG00000130201	*EXOC3L2*
				ENSG00000007047	*MARK4*
chr19	48,945,113	− 1.67386	5.63E−05	ENSG00000105464	*GRIN2D*
chr19^*^	45,721,139	− 0.36067	6.24E−05	ENSG00000130201	*EXOC3L2*
				ENSG00000007047	*MARK4*
chr22	50,616,743	2.576796	6.52E−05	ENSG00000073150	*PANX2*
chr12	132,922,443	− 0.85158	6.6E−05		
chr13	114,322,962	− 1.33518	6.9E−05	ENSG00000185974	*GRK1*
chr22	50,616,740	2.562951	6.92E−05	ENSG00000073150	*PANX2*
chr19	48,945,126	− 1.64199	7.32E−05	ENSG00000105464	*GRIN2D*
chr22	50,616,733	2.538738	7.84E−05	ENSG00000073150	*PANX2*
chr19	48,945,131	− 1.63696	8.05E−05	ENSG00000105464	*GRIN2D*
chr16^*^	1,835,849	− 1.47921	8.48E−05	ENSG00000095906	*NUBP2*
				ENSG00000162032	*SPSB3*
chr6	41,207,271	0.322382	9.19E−05	ENSG00000212176	*RNA5SP207*
FVC					
chr2	227,662,476	2.083124	4.88E−06	ENSG00000169047	*IRS1*
chr2	227,662,482	2.066378	5.49E−06	ENSG00000169047	*IRS1*
chr2	227,662,501	2.013397	6.8E−06	ENSG00000169047	*IRS1*
chr1	3,329,105	0.283266	9.86E−06	ENSG00000142611	*PRDM16*
chr2	242,955,278	− 3.46707	1.22E−05	ENSG00000233806	*LINC01237*
chr7	56,243,280	− 0.31459	1.51E−05		
chr1	40,388,312	0.248359	1.64E−05		
chr1	34,090,712	0.342628	1.72E−05	ENSG00000121904	*CSMD2*
chr2	227,662,462	1.963614	1.82E−05	ENSG00000169047	*IRS1*
chr9	34,809,867	− 0.32198	1.94E−05		
chr1	212,456,833	− 1.22998	2E−05	ENSG00000226251	*LINC02608*
chr2	227,662,459	1.962235	2.04E−05	ENSG00000169047	*IRS1*
chr1	34,090,722	0.360194	2.13E−05	ENSG00000121904	*CSMD2*
chr1	40,388,299	0.247353	2.44E−05		
chr4	39,719,509	− 0.33384	2.57E−05	ENSG00000078140	*UBE2K*
chr4	39,719,504	− 0.33124	2.65E−05	ENSG00000078140	*UBE2K*
chr1	45,203,996	0.682629	2.67E−05		
chr11	65,547,072	− 0.2697	2.84E−05	ENSG00000254470	*AP5B1*
chr2	227,662,443	1.966677	3.2E−05	ENSG00000169047	*IRS1*
chr19	55,881,590	1.590547	3.51E−05	ENSG00000095752	*IL11*
chr2	227,662,433	1.985295	3.55E−05	ENSG00000169047	*IRS1*
chr2	227,662,426	2.006028	3.6E−05	ENSG00000185950	*IRS2*
chr14	94,405,044	− 0.85692	3.86E−05	ENSG00000100628	*ASB2*
chr17	79,067,393	2.782969	3.97E−05	ENSG00000175866	*BAIAP2*
chr19	55,881,582	1.540431	4.08E−05	ENSG00000095752	*IL11*
chr7	56,243,259	− 0.2817	4.14E−05		
chr9	34,809,843	− 0.27944	4.38E−05		
chr17	75,613,156	0.367264	4.43E−05		
chr16	2,301,960	− 1.99883	4.49E−05	ENSG00000167969	*ECI1*
chr5	179,554,467	0.172032	5.14E−05	ENSG00000146090	*RASGEF1C*
chr9	34,809,878	− 0.33026	5.28E−05		
chr17	75,613,186	0.259034	5.33E−05		
chr12	106,461,103	− 1.84659	5.55E−05	ENSG00000074590	*NUAK1*
chr16	2,301,969	− 1.97289	5.66E−05	ENSG00000167969	*ECI1*
chr7	56,243,241	− 0.27378	5.66E−05		
chr2	227,662,390	2.353652	5.79E−05	ENSG00000169047	*IRS1*
chr5	179,554,462	0.167814	5.91E−05	ENSG00000146090	*RASGEF1C*
chr7	56,243,233	− 0.2686	5.96E−05		
chr13^*^	114,525,556	− 2.57251	6.25E−05	ENSG00000183087	*GAS6*
				ENSG00000233695	*GAS6 − AS1*
chr5	179,554,486	0.182607	6.36E−05	ENSG00000146090	*RASGEF1C*
chr2	72,359,706	0.217285	6.44E−05	ENSG00000003137	*CYP26B1*
chr19	22,883,687	2.353067	7E−05		
chr1	40,388,332	0.260153	7.03E−05		
chr20	62,188,249	0.178865	7.56E−05		
chr19	22,883,684	2.335013	7.71E−05		
chr19	36,757,583	0.700594	7.8E−05		
chr20	62,188,262	0.179876	8.09E−05		
chr22	50,758,097	0.338362	8.16E−05	ENSG00000205593	*DENND6B*
chr12	123,750,717	− 0.26888	8.23E−05	ENSG00000111328	*CDK2AP1*
chr7	2,106,405	− 3.75532	8.27E−05	ENSG00000002822	*MAD1L1*
chr7	56,243,224	− 0.25655	8.39E−05		
chr4	39,719,480	− 0.31037	8.63E−05	ENSG00000078140	*UBE2K*
chr1	181,382,667	0.338413	8.9E−05	mRNA	*AF387615*
chr4	39,719,523	− 0.34507	9.3E−05	ENSG00000078140	*UBE2K*
chr10	88,702,832	− 1.01265	9.46E−05	ENSG00000173269	*MMRN2*
chr2	72,359,687	0.201322	9.56E−05	ENSG00000003137	*CYP26B1*
chr16	86,528,639	− 1.92693605	2.2733E−06	ENSG00000268388	*FENDRR*
chr11	89,900,493	− 8.57710522	1.042E−05	ENSG00000077616	*NAALAD2*
chr6	168,708,413	2.6198784	1.0891E−05	ENSG00000164488	*DACT2*
chr11	89,900,518	− 8.40993874	1.2848E−05	ENSG00000077616	*NAALAD2*
chr16	86,528,603	− 2.37934592	1.4343E−05	ENSG00000268388	*FENDRR*
chr16	86,528,620	− 2.56207502	1.4967E−05	ENSG00000268388	*FENDRR*
chr2	233,791,733	2.516547	1.601E−05	ENSG00000066248	*NGEF*
chr16	86,528,600	− 2.29834345	1.6392E−05	ENSG00000268388	*FENDRR*
chr6	168,708,422	2.86106788	1.7273E−05	ENSG00000164488	*DACT2*
chr11	130,491,262	− 3.95462385	2.2669E−05		
chr6	168,708,401	2.15447478	2.3564E−05	ENSG00000164488	*DACT2*
chr2	233,791,742	2.27761208	3.1105E−05	ENSG00000066248	*NGEF*
chr16	86,528,611	− 2.78067202	3.1934E−05	ENSG00000268388	*FENDRR*
chr11	130,491,234	− 3.50377946	3.2327E−05		
chr11	130,491,229	− 3.49295666	3.2607E−05		
chr11	130,491,225	− 3.49165833	3.3003E−05		
chr11	130,491,218	− 3.4800439	3.9356E−05		
chr11	130,491,274	− 5.32113664	4.9746E−05		
chr20	61,992,129	8.76860666	5.2202E−05	ENSG00000101204	*CHRNA4*
chr11	1,103,266	− 1.68641265	5.3477E−05	ENSG00000198788	*MUC2*
chr4	10,508,681	− 3.48381736	5.411E−05	ENSG00000109684	*CLNK*
chr11	130,491,277	− 5.2862281	5.6835E−05		
chr8	6,671,626	− 2.57808863	5.7509E−05	ENSG00000275591	*XKR5*
chr1	7,022,170	8.69574765	5.8044E−05	ENSG00000171735	*CAMTA1*
chr11	1,103,270	− 1.68935565	5.9685E−05	ENSG00000198788	*MUC2*
chr4	190,537,048	− 3.24980484	7.2585E−05		
chr14	104,642,230	− 4.53713759	7.4715E−05	ENSG00000066735	*KIF26A*
chr4	190,537,044	− 3.22472764	7.9082E−05		
chr5	28,928,500	− 8.72472679	8.3633E−05		
chr16	86,528,570	− 1.82984517	8.7526E−05	ENSG00000268388	*FENDRR*
chr22	29,075,315	11.8597904	9.15E−05	ENSG00000100154	*TTC28*

^*^The CpG sites were annotated to more than one gene

A total of 280 common CpG sites (*P* < 0.05) were found for FEV1, FVC, and FEV1/FVC. 794 common genes (*P* < 0.05) were found for FEV1, FVC, and FEV1/FVC, among which two genes reached the level of *P* < 1 × 10^–3^, including *CHRNA4* and *MAD1L1*.

### Biological pathway analysis

The number of genomic cis-regulatory regions related to one or more genes was 13,821, 14,901, and 17,929 for FEV1, FVC, and FEV1/FVC, respectively (Additional file 6: Fig. S4). The absolute distance of genomic regions to transcription start site was displayed in Additional file 7: Fig. S5 and Additional file 8: Fig. S6.

The analysis found 12 common functional clusters of biological process with very high statistical significance (binomial *p*-value < 1.07E−13) (Table 2), including negative regulation of phospholipid biosynthetic process, platelet-derived growth factor binding, potassium:chloride symporter activity, epithelial-mesenchymal cell signaling, decreased serum estradiol, low voltage-gated calcium channel activity, cAMP response element binding protein binding, activation of Cdc42 GTPase activity, ceramide signaling pathway, transcription regulation by bZIP transcription factor, mitogen-activated protein kinase p38 binding, and notch signaling pathway.

**Table 2:** Significant common functional clusters biological process related to pulmonary function by GREAT using binomial test

Term name	Binom raw *P*-value	Binom FDR Q-value	Binom fold enrichment	Binom expected region hits	Binom observed region hits
FEV1					
Negative regulation of phospholipid biosynthetic process	2.30E−112	2.40E−108	22.67703	5.159407	117
Platelet-derived growth factor binding	1.75E−66	6.45E−63	6.609811	21.48322	142
Potassium:chloride symporter activity	2.13E−59	2.62E−56	27.73878	2.018834	56
Epithelial-mesenchymal cell signaling	3.35E−57	3.18E−54	6.273671	20.24333	127
Decreased serum estradiol	2.18E−47	2.23E−44	21.93261	2.234116	49
Low voltage-gated calcium channel activity	1.91E−44	1.41E−41	18.17954	2.750345	50
cAMP response element binding protein binding	1.41E−38	5.76E−36	9.067368	7.058278	64
Activation of Cdc42 GTPase activity	3.30E−36	5.30E−34	12.54469	3.906035	49
Ceramide signaling pathway	3.85E−33	1.69E−30	2.874993	61.21754	176
Transcription regulation by bZIP transcription factor	2.38E−29	3.61E−27	3.509946	33.04894	116
Mitogen-activated protein kinase p38 binding	9.76E−16	5.81E−14	6.090298	5.418454	33
Notch signaling pathway	3.93E−13	9.97E−12	2.123615	54.15295	115
FVC					
Activation of Cdc42 GTPase activity	5.16E−82	5.39E−78	20.93006	4.204478	88
Potassium:chloride symporter activity	1.08E−73	1.99E−70	30.83174	2.173085	67
Negative regulation of phospholipid biosynthetic process	1.82E−54	5.77E−52	13.14459	5.553615	73
Epithelial-mesenchymal cell signaling	1.61E−45	3.29E−43	5.323534	21.79004	116
Decreased serum estradiol	2.81E−43	1.92E−40	19.54412	2.404816	47
Platelet-derived growth factor binding	3.14E−36	1.05E−33	4.583852	23.12466	106
Low voltage-gated calcium channel activity	1.08E−35	3.33E−33	14.86242	2.960487	44
Transcription regulation by bZIP transcription factor	1.57E−31	2.39E−29	3.513795	35.57407	125
Notch signaling pathway	4.40E−29	1.16E−26	2.856257	54.26683	155
cAMP response element binding protein binding	1.22E−19	1.05E−17	5.791325	7.597571	44
Mitogen-activated protein kinase p38 binding	3.38E−17	2.04E−15	6.172357	5.832456	36
Ceramide signaling pathway	1.07E−13	4.56E−12	2.033541	65.89492	134
FEV1/FVC					
Negative regulation of phospholipid biosynthetic process	7.31E−162	7.63E−158	24.44504	6.708927	164
Platelet-derived growth factor binding	3.83E−103	1.41E−99	7.338399	27.93525	205
Epithelial-mesenchymal cell signaling	2.93E−64	1.18E−61	5.812409	26.32299	153
Activation of Cdc42 GTPase activity	3.00E−58	8.25E−56	14.56942	5.079131	74
Low voltage-gated calcium channel activity	9.99E−55	6.14E−52	17.61571	3.576354	63
Ceramide signaling pathway	5.35E−44	3.53E−41	2.914465	79.60295	232
Potassium:chloride symporter activity	7.68E−43	1.89E−40	18.28468	2.625149	48
Decreased serum estradiol	5.03E−36	1.55E−33	15.14585	2.905086	44
cAMP response element binding protein binding	9.55E−35	1.76E−32	7.299999	9.178084	67
Notch signaling pathway	4.98E−31	9.39E−29	2.730495	65.55589	179
Transcription regulation by bZIP transcription factor	2.74E−27	4.16E−25	3.04832	42.9745	131
Mitogen-activated protein kinase p38 binding	1.19E−21	7.41E−20	6.386807	7.045774	45

The MSiDB and PANTHER pathway, Human Phenotype, and Go enriched terms of FEV1, FVC, and FEV1/FVC are shown in Additional file 9: Table S3, Additional file 10: Table S4, and Additional file 11: Table S5, respectively.

### Region-based analysis

By using *comb-p*, region-based analyses identified 13, 14, and 12 DMRs (FDR < 0.05) associated with FEV1, FVC, and FEV1/FVC ratio, respectively (Table 3). Interestingly, 4 significant FEV1 associated DMRs (from 41,207,271 to 41,207,436 bp and from 43,394,513 to 43,394,685 bp on chromosome 6; from 50,616,620 to 50,617,148 bp on chromosome 22; from 40,996,995 to 40,997,142 bp on chromosome 17), 2 significant FVC associated DMRs (from 179,554,269 to 179,554,550 bp on chromosome 5; from 39,719,381 to 39,719,533 bp on chromosome 4), and 1 significant FEV1/FVC ratio associated DMR (from 130,491,143 to 130,491,278 bp on chromosome 11) cover the corresponding top significant CpG sites in Table 1.

**Table 3:** The results of annotation to the significant DMRs (slk corrected *P*-value < 0.05)

Chromosome	Start	End	Length	Stouffer-liptak-kechris(slk) corrected *P*-value	Ensembl ID	Gene symbol
FEV1						
chr6	41,207,271	41,207,436	10	0.001728		
chr6	106,553,539	106,553,708	11	0.001782	ENSG00000057657	*PRDM1*
chr18	46,502,900	46,503,123	14	0.004627		
chr6	43,394,513	43,394,685	12	0.006354		
chr1	43,814,661	43,814,895	21	0.008471	ENSG00000117400	*MPL*
chr14	24,780,505	24,780,906	12	0.009266	ENSG00000136305	*CIDEB*
					ENSG00000213906	*LTB4R2*
chr11	20,626,786	20,627,432	26	0.01328	ENSG00000165970	*SLC6A5*
chr2	175,205,113	175,205,752	29	0.01328		
chr3	184,294,568	184,294,844	12	0.01516	ENSG00000182580	*EPHB3*
chr22	50,616,620	50,617,148	29	0.01753	ENSG00000114735	*HEMK1*
chr2	39,470,838	39,471,149	34	0.03594	ENSG00000205111	*CDKL4*
chr1	247,463,964	247,464,319	21	0.03833	ENSG00000162714	*ZNF496*
chr17	40,996,995	40,997,142	11	0.0463	ENSG00000131480	*AOC2*
FVC						
chr5	179,554,269	179,554,550	22	0.003109	ENSG00000146090	*RASGEF1C*
chr4	39,719,381	39,719,533	10	0.004208	ENSG00000078140	*UBE2K*
chr6	41,207,271	41,207,436	10	0.007586	ENSG00000212176.1	*RNA5SP207*
chr19	4,792,661	4,793,200	24	0.009082	ENSG00000141965	*FEM1A*
chr6	106,553,539	106,553,708	11	0.009722	ENSG00000057657	*PRDM1*
					ENSG00000057663	*ATG5*
chr1	43,814,661	43,814,895	21	0.01655	ENSG00000117400	*MPL*
chr6	41,650,731	41,651,148	26	0.01748		
chr3	184,294,568	184,294,844	12	0.02198	ENSG00000182580	*EPHB3*
chr2	176,931,544	176,931,983	16	0.02289		
chr2	39,470,838	39,471,149	34	0.02463		
chr18	14,998,779	15,000,083	61	0.04006		
chr5	134,744,537	134,744,742	14	0.04018		
chr1	148,902,200	148,902,378	15	0.04418		
chr2	91,874,335	91,874,482	12	0.04763	ENSG00000175658	*DRD5P2*
FEV1/FVC						
chr15	68,115,731	68,116,609	24	0.002354	ENSG00000188779	*SKOR1*
chr11	130,491,143	130,491,278	10	0.00646		
chr9	128,985,373	128,985,521	11	0.007998		
chr20	25,990,367	25,990,728	18	0.008941		*LOC100134868*
chr16	895,385	895,537	11	0.01412		
chr9	124,308,098	124,308,286	11	0.01419		
chr3	22,458,309	22,458,548	13	0.01914		
chr15	68,549,191	68,549,322	9	0.01938	ENSG00000128973	*CLN6*
chr7	329,073	330,975	103	0.02247		*LOC100288524*
chr17	80,840,674	80,841,003	18	0.02549	ENSG00000141556	*TBCD*
chr17	75,525,368	75,525,475	4	0.03143	ENSG00000267665	*LOC400622*
chr19	1,229,184	1,230,113	60	0.04449	ENSG00000099625	*CBARP*

Of all DMRs, three DMRs (located at *PRDM1*, *MPL*, *EPHB3*) were related to more than one trait. Of the significant DMRs associated with pulmonary function, nine DMRs for FEV1 were annotated to *PRDM1* on chromosome 6, *MPL* and *ZNF496* on chromosome 1, *CIDEB* and *LTB4R2* on chromosome 14, *SLC6A5* on chromosome 11, *EPHB3* on chromosome 3, *HEMK1* on chromosome 22, *CDKL4* on chromosome 2, and *AOC2* on chromosome 17. Nine DMRs for FVC were annotated to *RASGEF1C* on chromosome 5, *UBE2K* on chromosome 4, *RNA5SP207*, *PRDM1*, and *ATG5* on chromosome 6, *FEM1A* on chromosome 19, *MPL* on chromosome 1, *EPHB3* on chromosome 3 and *DRD5P2* on chromosome 2. And seven DMRs for FEV1/FVC ratio were annotated to *RASGEF1C* on chromosome 5, *UBE2K* on chromosome 4, *RNA5SP207*, *PRDM1*, and *ATG5* on chromosome 6, *FEM1A* on chromosome 19, *MPL* on chromosome 1, *EPHB3* on chromosome 3 and *DRD5P2* on chromosome 2. In addition, four DMRs for FEV1, six DMRs for FVC, and five DMRs for FEV1/FVC ratio were located in the intergenic regions.

**Fig. 2:**
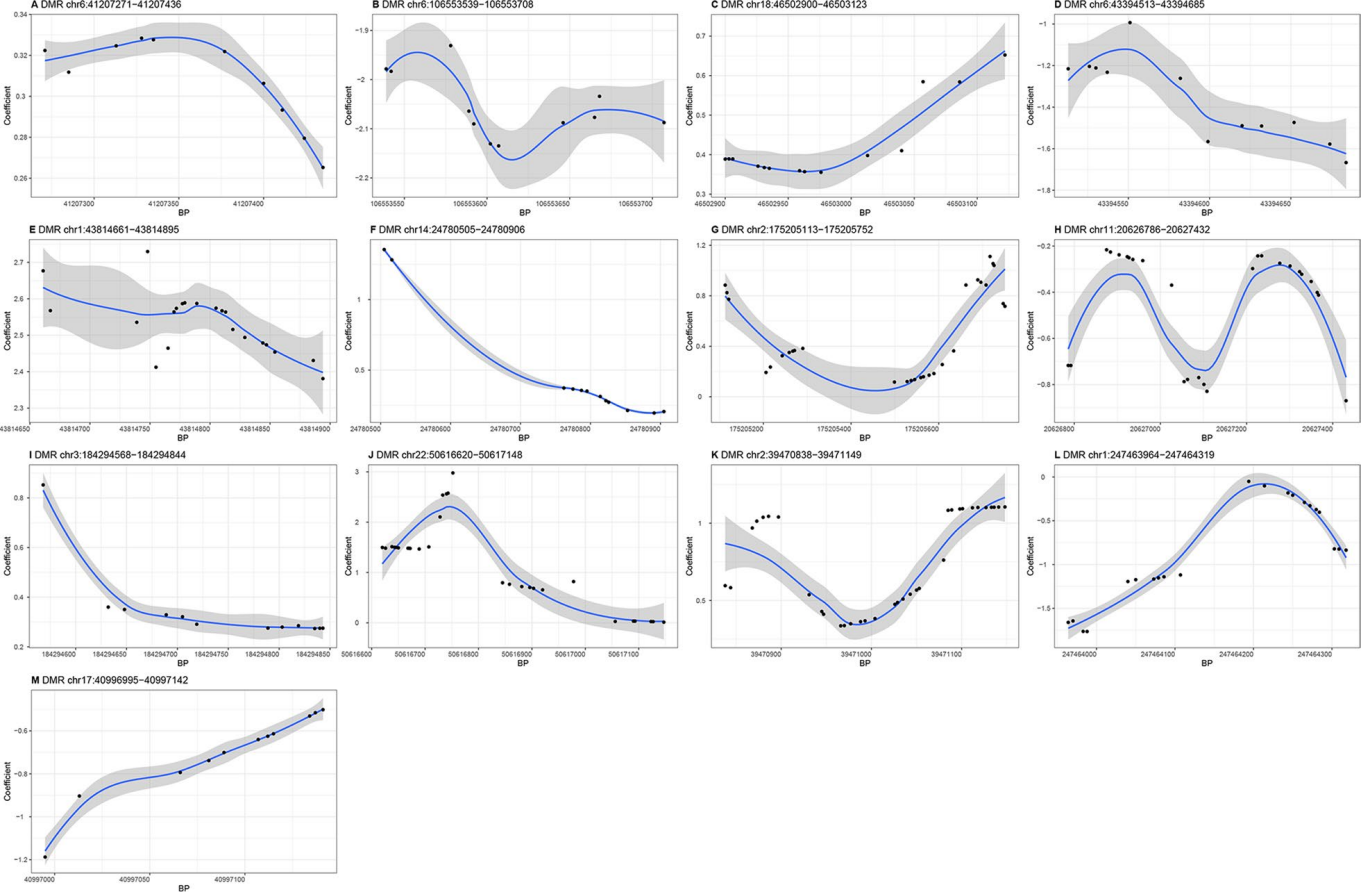
Differential methylation patterns for FEV1 from the 13 DMRs. Except the three DMRs (**G**, **J**, **L**), nine DMRs (**A**, **C**, **E**, **F**, **I**, **K**) were positively and four DMRs (**B**, **D**, **H**, **M**) negatively associated with FEV1

**Fig. 3:**
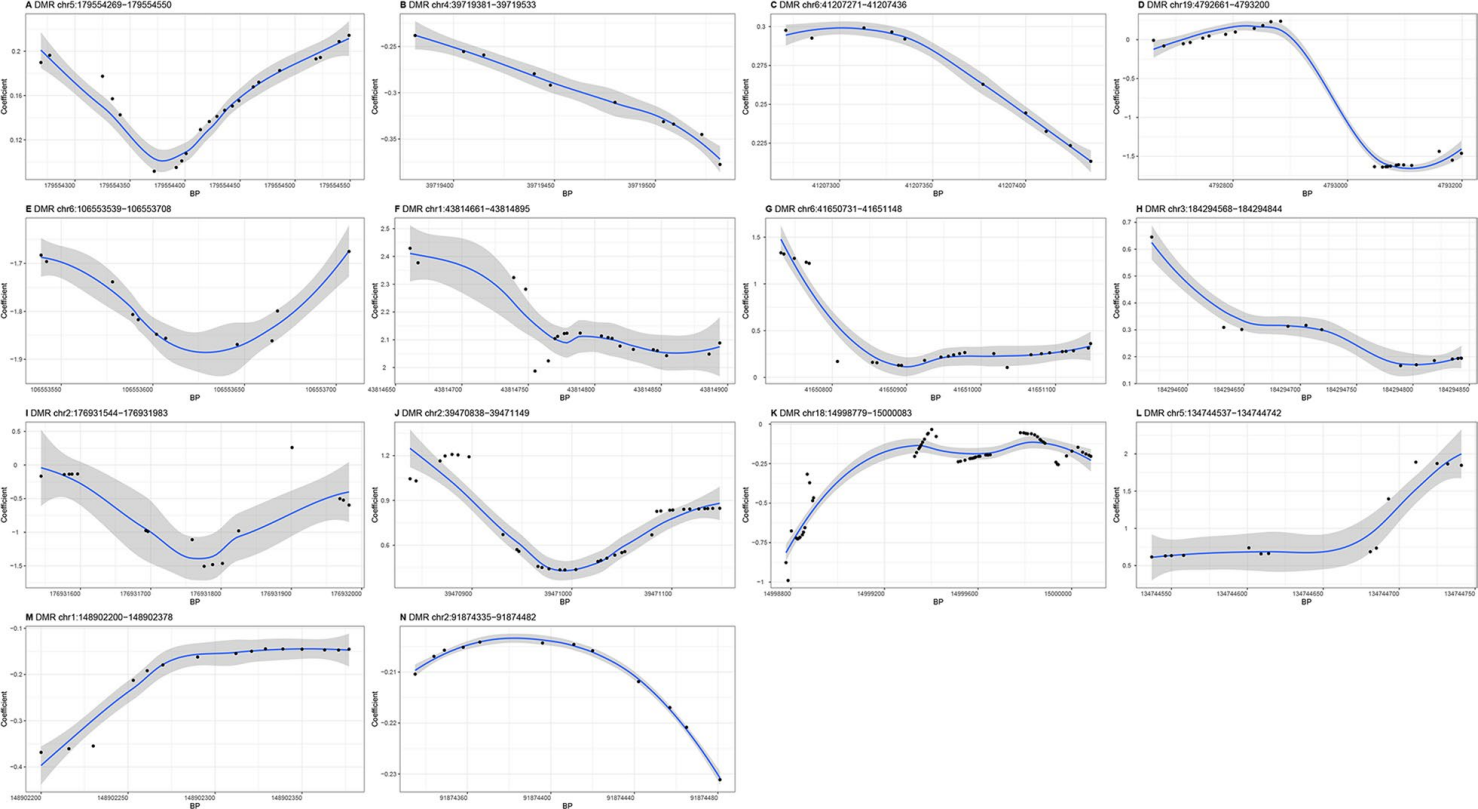
Differential methylation patterns for FVC from the 14 DMRs. Except the two DMRs (**D**, **I**), seven DMRs (**A**, **C**, **F**, **G**, **H**, **J**, **L**) were positively and five DMRs (**B**, **E**, **K**, **M**, **N**) negatively associated with FVC

**Fig. 4:**
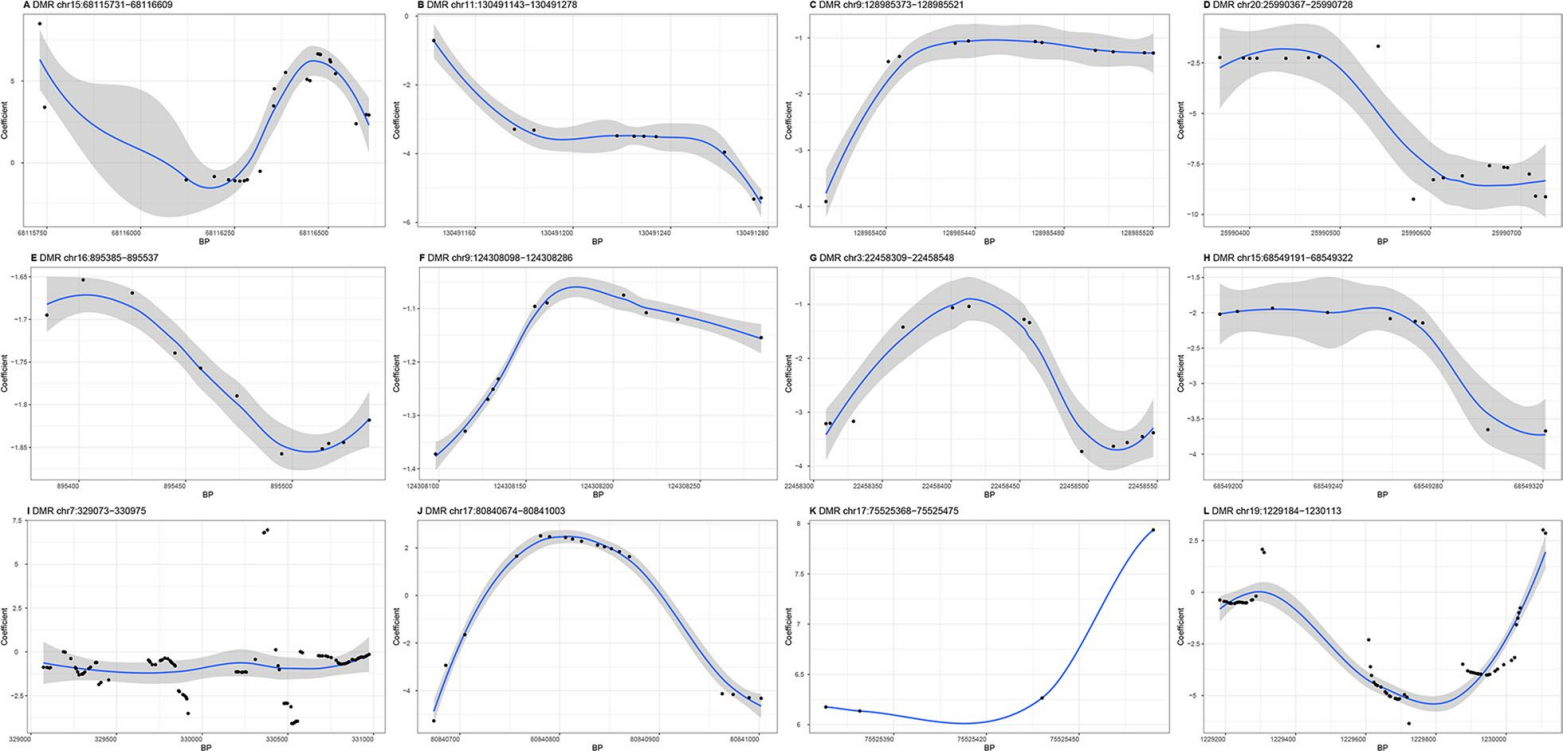
Differential methylation patterns for FEV1/FVC from the 12 DMRs. Except the four DMRs (**A**, **I**, **J**, **L**), one DMRs (**K**) was positively and seven DMRs (**B**, **C**, **D**, **E**, **F**, **G**, **H**) negatively associated with FEV1/FVC ratio

Figures 2, 3 and 4 display the methylation patterns for the significant DMRs for pulmonary function in Table 3, of which six DMRs (A, C, E, F, I, K) were positively and four DMRs (B, D, H, M) negatively associated with FEV1, and seven DMRs (A, C, F, G, H, J, L) were positively and five DMRs (B, E, K, M, N) negatively associated with FVC. One DMRs (K) was positively and seven DMRs (B, C, D, E, F, G, H) negatively associated with FEV1/FVC ratio.

### Gene expression analysis

In the gene expression analyses, we included 12 twin pairs (7 male pairs) with median age of 53 years (ranging from 43 to 65), a median FEV1 of 2.05 (ranging from 1.04 to 3.81), a median FVC of 2.17 (ranging from 1.32 to 4.10), and a median FEV1/FVC of 0.97(ranging from 0.57 to 1.01).

### Weighted gene co-expression network analysis (WGCNA)

As shown in Additional file 12: Fig. S7, the genes clustered in lightsteelblue1 module (including 492 genes) were both positively correlated with FEV1 (*r* = 0.58, *P* = 0.003) and FVC (*r* = 0.51, *P* = 0.01). The genes clustered in this module were significantly enriched in positive regulation of protein secretion, positive regulation of cell division, growth factor activity, calcium ion binding, motile cilium, platelet degranulation, and phospholipase A2 activity. (Additional file 13: Table S6).

Moreover, the genes clustered in darkorange2 module (including 62 genes) were also both positively correlated with FEV1(*r* = 0.45, *P* = 0.03) and FVC (*r* = 0.53, *P* = 0.007). The genes clustered in this module were significantly enriched in extracellular region, negative regulation of exocytosis, and cell adhesion (Additional file 14: Table S7).

Additionally, the genes clustered in ivory module (including 76 genes) were negatively correlated with FEV1/FVC (r = − 0.63, *P* = 0.001). The genes clustered in this module were significantly enriched in cytokine activity, extracellular region, intermediate filament, and so on (Additional file 15: Table S8).

### The common genes and enrichment terms between methylation analysis and WGCNA

We detected the common genes and enrichment terms between the methylation analyses and WGCNA. We found *DIP2C* gene which included in lightsteelblue1 modules linked to FEV1, and *ASB2* which included in darkorange2 modules associated with FVC. The common enrichment terms “platelet alpha granule lumen” was identified.

### Correlation analysis

Significant correlations between gene expression levels and pulmonary function indices were identified, including SLC6A5 related to FEV1 (*r* = 0.454, *P* = 0.026), and GAS6 related to FVC (*r* = 0.533, *P* = 0.007).

## Discussion

In this study, we detected the epigenetic variants of pulmonary function using EWAS based on monozygotic twin design. The number of CpG sites which was identified to reached the level of *P* < 1 × 10^–4^ was 25 for FEV1, 56 for FVC, and 31 for FEV1/FVC. And 12 significant pathways of interest for pulmonary function were highlighted by GREAT ontology enrichment analyses. Finally, we identified several DMRs related to pulmonary function, and of all DMRs, three (*PRDM1*, *MPL*, and *EPHB3*) were related to more than one trait. Two genes (*DI92C* and *ASB2*) and one enrichment terms (platelet alpha granule lumen) were overlapped between methylation analysis and WGCNA. Finally, two genes were found to be correlated to pulmonary function.

The genes *DIP2C*, *WDR90, PANX2*, *NUBP2*, *AP5B1*, *CYP26B1*, *GAS6*, *IL11*, *IRS1*, *IRS2*, *MAD1L1*, *CAMTA1*, *CHRNA4*, *FENDRR*, *MUC2* associated with top CpG sites (Table 1) played important roles in pulmonary function. Most interestingly, *DIP2C* gene was not only identified to link to pulmonary function in our EWAS results, but further validated in the WGCNA. Moreover, *DIP2C* has been detected to related to pulmonary function in blood DNA in Koreans adults [34]. Mutations in *DIP2C* have been identified in lung cancer samples [35]. This demonstrated that *DIP2C* gene indeed plays an important role at the pulmonary disease. *WDR90* was identified as required gene for ciliogenesis [36]. The lung ciliary-related proteins keeping the airways clear of mucus and dirt play a role in human pulmonary function. *PANX2* was expressed in human airway epithelial cells and alveolar macrophages, which might have an impact on pulmonary function [37]. *NUBP2* was found to express in distal lung epithelium, which might function in lung development of mice [38]. *AP5B1* was identified as susceptibility loci for the combined eczema plus asthma phenotype, which might affect pulmonary function [39]. *Cyp26b1* was an essential regulator of distal airway epithelial differentiation during lung development [40]. *GAS6* promoted Axl-mediated survival in pulmonary endothelial cells [41]. *IL-11* was suggested that could cause lung inflammation and airway obstruction [42]. *IRS1* and *IRS2* were found to mediate *IL-4*-induced migration of human airway epithelial cells, which influence pulmonary function [43]. *MAD1L1* was identified as a genome-wide significant signals with idiopathic pulmonary fibrosis by GWAS [44]. *CAMTA1* was a regulator of nuclear factor of activated T cells signaling, which was linked to pulmonary arterial hypertension [45]. *FENDRR* was long noncoding RNA exhibiting antifibrotic activity in pulmonary fibrosis [46]. Decreased expression of *MUC2* has been observed in patients with COPD [47].

Pathway enrichment analyses showed lots of common significant pathways of pulmonary function using GREAT. The significant enrichment pathway include negative regulation of phospholipid biosynthetic process [48], platelet-derived growth factor binding [49], potassium:chloride symporter activity [50], epithelial-mesenchymal cell signaling [51], decreased serum estradiol [52], low voltage-gated calcium channel activity [53], cAMP response element binding protein binding [54], activation of Cdc42 GTPase activity [55], ceramide signaling pathway [56], transcription regulation by bZIP transcription factor [57], mitogen-activated protein kinase p38 binding [58], and notch signaling pathway [59].

The genomic region-based analyses found 39 DMRs locating at 23 genes (Table 3), of which *PRDM1*, *MPL*, *LTB4R2*, *EPHB3* and *SLC6A5* had certain biological function potentially linked to pulmonary function. Previous study found that NF-κB(p65) promotion of miR-99b could aggravate acute lung injury by *PRDM1* down-regulation, and over-expressed *PRDM1* inhibits acute lung injury in mice [60]. *MPL* was defined as an important gene in a novel VEGF–miR-1–Mpl–P-selectin effector pathway in lung Th2 inflammation and found as potential therapeutic targets for asthma [61]. *LTB4R2*, as one of pivotal leukotriene B4 receptors, was proposed as potential therapeutic targets in asthma [62]. *EphB3* was expressed at human lung fibroblasts, which induce dephrin-B2 forward signal involved in several fibroblast functions [63]. *SLC6A5*, also named *GLYT-2*, encoded a sodium- and chloride-dependent glycine neurotransmitter transporter. The glycinergic inhibitory synaptic inputs played an important role in respiratory motoneurons, which could affect pulmonary function [64].

As additional validation, we integrated the methylation data with gene expression data. Genes clustered in lightsteelblue1 and darkorange2 modules were positively correlated with FEV1 and FVC in WGCNA, and some genes were in common with EWAS findings, including *DIP2C* discussed above and *ASB2* involved in pulmonary function remained to be studied further. Additionally, *SLC6A5* and *GAS6* discussed above were positively correlated to pulmonary function. Moreover, the common enrichment terms between methylation analysis and WGCNA was platelet alpha granule lumen, which involved in pulmonary function remained to be studied further.

There were several strengths in the present study. The identical twin design used in our study to detect the epigenetic variation of pulmonary function could perfectly control over the genetic background to provide credible results. Moreover, this was one of the few pulmonary function EWA studies in Asian and the first in Chinese. As the genetic background and environmental exposures differ from ethnic populations, our study elucidated the underlying physiological mechanism of pulmonary function changes in Chinese adults. However, our studies also have some limitations. First, compared with other general case–control design, the sample size of our study was relatively small due to the difficulty of recruiting and identifying qualified MZ twin pairs. However, previous study has determined that the sample sizes of monozygotic twins just require roughly 1/4 of sample sizes in the ordinary case-only design to provide the sufficient power [65]. Second, the DNA sample was extracted from blood rather than the lung tissue. Although we know methylation is the characteristic of tissue-specificity, it was difficult to obtain the lung tissue of sample. Moreover, the mounting evidences have supported disease-associated methylation loci could be identified from peripheral samples [66]. Third, the non-shared environment for the individual siblings of MZ twins, such as occupational environment [67], residential environment [68], and mode of transport [69], could expose themselves to different levels of environmental pollutants, including particulate matter, nitrogen dioxide; volatile organic compounds, polycyclic aromatic hydrocarbons, and so on, which might directly affect pulmonary function [70–73], and cause different levels of DNA methylation [74–78] thereby indirectly influencing pulmonary function. However, due to the complicated causes of DNA methylation and the difficulty of monitoring for the external environmental exposure, we have not further analyzed the causes of DNA methylation. We will seek practical method to solve it in the future research.

Although these results could not immediately be applied as clinical predictors of disease in individuals, they are important from an aetiological perspective. Epigenetic studies complement genetic association studies to identify pulmonary function related genes. The EWAS and gene expression analysis identified candidate genes and pathways related to pulmonary function, which could help understand underlying mechanisms of pulmonary function and explore new molecular biological pathway of pulmonary functional decline in clinical.

## Conclusion

In conclusion, our DNA methylation sequencing analysis on identical twins provides new references for the epigenetic regulation on pulmonary function. Several CpG sites, genes, biological pathways and DMRs were considered as possible crucial to pulmonary function. All findings point important clues to further explore of pulmonary function.

## Supplementary Information

**Additional file 1: Table S1. **Descriptive statistics of basic characteristicsof the sample.

**Additional file 2: TableS2.** Descriptive statistics of intra-pair difference of FEV1, FVC, FEV1/FVCratio and some significant methylated value of CpG sites.

**Additional file 3: Figure S1.** Scatter plots with regression lineshowing the association of Δ methylated value of CpG sites and Δ FEV1. The Δmethylation value of four CpG sites (f, h, i, j) were positively correlatedwith ΔFEV1, and the Δ methylation value of seven CpG sites (a,b,c,d,e,g,k) werenegatively correlated with ΔFEV1.

**Additional file 4: Figure S2.** Scatter plot with regression line showing the association of Δ methylatedvalue of CpG sites and Δ FVC. The Δ methylation value of eleven CpG sites(a,b,c,g,h,i,j,k,m,o,q) were positively correlated with ΔFVC, and the Δmethylation value of six CpG sites (d,e,f,l,n,p) were negatively correlatedwith ΔFVC.

**Additional file 5: Figure S3.** Scatter plots with regression line showing the association of Δ methylatedvalue of CpG sites and Δ FEV1/FVC. The Δ methylation value of two CpG sites(c,i) were positively correlated with ΔFEV1/FVC ratio, and the Δ methylation valueof ten CpG sites (a,b,d,e,f,g,h,j,k,l) were negatively correlated withΔFEV1/FVC ratio.

**Additional file 6: Figure S4.** Number of associated genes per region for FEV1 (a), FVC (b), and FEV1/FVC (c). The number of genomiccis-regulatory regions related with one or more genes was 13,821 for FEV1,14,901 for FVC, and 17,929 for FEV1/FVC.

**Additional file 7: Figure S5.** Binned by orientation and distance to transcription start site FEV1 (a),FVC (b), and FEV1/FVC (c). Thenumber of FEV1 genomic regions whose distance to the TSS was <-500, -500 to-50, -50 to -5, -5 to 0, 0 to 5, 5 to 50, 50 to 500, >500 kb was 478, 3691,3732, 1666, 2440, 5817, 6066, and 632, respectively. The number of FVC genomicregions whose distance to the TSS was <-500, -500 to -50, -50 to -5, -5 to0, 0 to 5, 5 to 50, 50 to 500, >500 kb was 549, 4046, 4054, 1816, 2666,6306, 6425, and 673, respectively. The number of FEV1/FVC genomic regions whosedistance to the TSS was <-500, -500 to -50, -50 to -5, -5 to 0, 0 to 5, 5 to50, 50 to 500, >500 kb was 610, 4826, 4673, 2537, 3125, 7458, 7530, and 840,respectively.

**Additional file 8: Figure S6.** Binned by absolute distance to transcription start site for FEV1 (a), FVC(b), and FEV1/FVC (c). Thenumber of FEV1 genomic regions whose absolute distance to the TSS was 0 to 5, 5to 50, 50 to 500, >500 kb was 4106, 9549, 9757, 1110, respectively. Thenumber of FVC genomic regions whose absolute distance to the TSS was 0 to 5, 5to 50, 50 to 500, >500 kb was 4482, 10360, 10471, 1222, respectively. Thenumber of FEV1/FVC genomic regions whose absolute distance to the TSS was 0 to5, 5 to 50, 50 to 500, >500 kb was 5662, 12131, 12356, 1450, respectively.

**Additional file 9: Table S3.** Significantfunctional clusters biological process related to FEV1 by GREAT using binomialtest.

**Additional file 10: Table S4.** Significantfunctional clusters biological process related to FVC by GREAT using binomialtest

**Additional file 11: Table S5.** Significantfunctional clusters biological process related to FEV1/FVC by GREAT usingbinomial test.

**Additional file 12: Figure S7.** Relationships of consensus module eigengenes and external traits. Each row inthe table corresponds to a consensus module, and each column to a sample ortrait. Numbers in the table report the correlations of the corresponding moduleeigengenes and traits, with the *P*-valuesprinted below the correlations in parentheses. The table is color coded bycorrelation according to the color legend. The genes clustered inlightstellblue1 module and darkorange2 module are the positively correlatedwith FEV1 and FVC, and the genes clustered in ivory module is negativelycorrelated with FEV1/FVC.

**Additional file 13: Table S6.** The results ofenrichment analysis for genes clustered in lightsteelblue1 module by DAVID tool.

**Additional file 14: Table S7.** The results ofenrichment analysis for genes clustered in darkorange2 module by DAVID tool

**Additional file 15: Table S8.** The results ofenrichment analysis for genes clustered in ivory module by DAVID tool.

## Data Availability

The datasets and/or analyzed during the current study are available from the corresponding authors on reasonable request.
