# Supplementary material for: Genome-wide DNA methylation analysis of pulmonary function in middle and old-aged Chinese monozygotic twins
Source: Respir Res. 2021 Nov 22;22:300. doi: 10.1186/s12931-021-01896-5 (PMC8609861; doi:10.1186/s12931-021-01896-5)
Supplement: Supplementary file 1 — Additional file 1: Table S1. Descriptive statistics of basic characteristicsof the sample. [file 12931_2021_1896_MOESM1_ESM.docx]

Table S1 Descriptive statistics of basic characteristics of the sample

|  | FEV1 | | |  | FVC | | |  | FEV1/FVC | | |
| --- | --- | --- | --- | --- | --- | --- | --- | --- | --- | --- | --- |
| Characteristics | Value | Intra-pair correlation | |  | Value | Intra-pair correlation | |  | Value | Intra-pair correlation | |
|  |  | r | *P*-value |  |  | r | *P*-value |  |  | r | *P*-value |
| Number of twin pairs | 60 |  |  |  | 59 |  |  |  | 44 |  |  |
| Gender, n (%) |  |  |  |  |  |  |  |  |  |  |  |
| Male | 34(56.7) |  |  |  | 34(57.6) |  |  |  | 21(47.7) |  |  |
| Female | 26(43.3) |  |  |  | 25(42.4) |  |  |  | 23(52.3) |  |  |
| Age, M (P_2.5_, P_97.5_), years | 51.5(40,66) |  |  |  | 51(40.0,66.0) |  |  |  | 50(39.23,64) |  |  |
| BMI, kg/m2, mean (SD) | 25.41(3.55) | 0.567 | <0.001 |  | 25.3(3.7) | 0.603 | <0.001 |  | 24.59(3.46) | 0.605 | <0.001 |
| Blood pressure |  |  |  |  |  |  |  |  |  |  |  |
| Systolic, M (P_2.5_, P_97.5_), mmHg | 130(105,187.92) | 0.348 | 0.006 |  | 130(103.95,188.17) | 0.381 | 0.003 |  | 129.5(102.45,193.42) | 0.282 | 0.064 |
| Diastolic, M (P_2.5_, P_97.5_), mmHg | 83.5(64,105.98) | 0.231 | 0.071 |  | 82(62,106.2) | 0.282 | 0.030 |  | 84(64.23,112.2) | 0.197 | 0.199 |
| SUA,μmol/L, M (P_2.5_, P_97.5_), | 286(153,548) | 0.369 | 0.003 |  | 285(152.25,550.6) | 0.389 | 0.003 |  | 274(141.38,497.25) | 0.475 | 0.001 |
| GLU, mmol/L, M (P_2.5_, P_97.5_), | 5.4(3.6,10.73) | 0.565 | <0.001 |  | 5.4(3.6,10.84) | 0.486 | <0.001 |  | 5.25(3.72,11.50) | 0.599 | <0.001 |
| CHOL, mmol/L, mean (SD) | 4.85(1.20) | 0.544 | <0.001 |  | 4.9(1.18) | 0.570 | <0.001 |  | 4.89(1.25) | 0.536 | <0.001 |
| TG, mmol/L, M (P_2.5_, P_97.5_), | 1.14(0.2,5.65) | 0.644 | <0.001 |  | 1.13(0.20,5.67) | 0.660 | <0.001 |  | 1.08(0.33,5.91) | 0.577 | <0.001 |
| HDLC, mmol/L, M (P_2.5_, P_97.5_), | 1.31(0.67,2.71) | 0.760 | <0.001 |  | 1.34(0.70,2.71) | 0.796 | <0.001 |  | 1.40(0.54,2.53) | 0.748 | <0.001 |
| LDL, mmol/L, mean (SD) | 2.83(0.91) | 0.486 | <0.001 |  | 2.83(0.89) | 0.465 | <0.001 |  | 2.86(0.92) | 0.505 | 0.001 |
| FEV1, mean (SD) | 1.98(0.72) | 0.556 | <0.001 |  |  |  |  |  |  |  |  |
| ΔFEV1, mean (SD) | 0.54(0.39) |  |  |  |  |  |  |  |  |  |  |
| ΔFEV1, %, M (P_2.5_, P_97.5_) | 0.12(0.03,0.38) |  |  |  |  |  |  |  |  |  |  |
| FVC, mean (SD) |  |  |  |  | 2.33(0.83) | 0.681 | <0.001 |  |  |  |  |
| ΔFVC, mean (SD) |  |  |  |  | 0.57(0.35) |  |  |  |  |  |  |
| ΔFVC, %, M (P_2.5_, P_97.5_) |  |  |  |  | 0.11(0.03,0.32) |  |  |  |  |  |  |
| FEV1/FVC, mean (SD) |  |  |  |  |  |  |  |  | 0.86(0.14) | -0.235 | 0.125 |
| ΔFEV1/FVC, mean (SD) |  |  |  |  |  |  |  |  | 0.18(0.12) |  |  |
| ΔFEV1/FVC, %, M (P_2.5_, P_97.5_) |  |  |  |  |  |  |  |  | 0.14(0.05,0.47) |  |  |

a. Continuous variables were presented as mean (standard deviation (SD)) or median (P_2.5_, P_97.5_); Categorical variables were presented as numbers with percentages.

BMI, body mass index; SUA, serum uric acid; GLU, fasting glucose; CHOL, total cholesterol; TG, triglyceride; HDLC, high-density lipoprotein cholesterol; LDLC, low-density lipoprotein cholesterol. FEV1: forced expiratory volume the first second; FVC, forced vital capacity; and FEV1/FVC: forced expiratory volume the first second/ forced vital capacity.
