# Supplementary material for: Genome-wide DNA methylation analysis of pulmonary function in middle and old-aged Chinese monozygotic twins
Source: Respir Res. 2021 Nov 22;22:300. doi: 10.1186/s12931-021-01896-5 (PMC8609861; doi:10.1186/s12931-021-01896-5)
Supplement: Supplementary file 2 — Additional file 2: TableS2. Descriptive statistics of intra-pair difference of FEV1, FVC, FEV1/FVCratio and some significant methylated value of CpG sites. [file 12931_2021_1896_MOESM2_ESM.docx]

Table S2. Descriptive statistics of intra-pair difference of FEV1, FVC, FEV1/FVC ratio and significant methylated value of CpG sites.

| Variables | Mean ± SD |
| --- | --- |
| ΔFEV1 | 0.10±0.66 |
| ΔMethylated value of CpG sites at chr3:138639540 | -0.42±4.58 |
| ΔMethylated value of CpG sites at chr3:138639552 | -0.37±4.60 |
| ΔMethylated value of CpG sites at chr3:138639544 | -0.40±4.59 |
| ΔMethylated value of CpG sites at chr3:138639520 | -0.50±4.55 |
| ΔMethylated value of CpG sites at chr6:43394632 | -0.13±3.01 |
| ΔMethylated value of CpG sites at chr16:706133 | 0.02±0.68 |
| ΔMethylated value of CpG sites at chr10:527775 | 0.41±3.31 |
| ΔMethylated value of CpG sites at chr22:50616743 | 0.68±5.16 |
| ΔMethylated value of CpG sites at chr22:50616740 | 0.68±5.17 |
| ΔMethylated value of CpG sites at chr22:50616733 | 0.67±5.20 |
| ΔMethylated value of CpG sites at chr16:1835849 | 0.83±3.74 |
|  |  |
| ΔFVC | 0.18±0.65 |
| ΔMethylated value of CpG sites at chr2:227662476 | 0.04±4.94 |
| ΔMethylated value of CpG sites at chr2:227662482 | 0.05±4.95 |
| ΔMethylated value of CpG sites at chr2:227662501 | 0.10±4.99 |
| ΔMethylated value of CpG sites at chr1:3329105 | 0.05±0.81 |
| ΔMethylated value of CpG sites at chr2:242955278 | -0.92±7.60 |
| ΔMethylated value of CpG sites at chr11:65547072 | 0.26±0.91 |
| ΔMethylated value of CpG sites at chr2:227662443 | 0.37±4.88 |
| ΔMethylated value of CpG sites at chr19:55881590 | -0.44±6.77 |
| ΔMethylated value of CpG sites at chr2:227662433 | 0.39±4.87 |
| ΔMethylated value of CpG sites at chr2:227662426 | 0.41±4.86 |
| ΔMethylated value of CpG sites at chr19:55881582 | -0.48±6.70 |
| ΔMethylated value of CpG sites at chr12:106461103 | 0.06±3.93 |
| ΔMethylated value of CpG sites at chr2:227662390 | 1.04±5.48 |
| ΔMethylated value of CpG sites at chr13:114525556 | -0.42±6.57 |
| ΔMethylated value of CpG sites at chr2:72359706 | 0.01±0.80 |
| ΔMethylated value of CpG sites at chr7:2106405 | -0.54±9.50 |
| ΔMethylated value of CpG sites at chr2:72359687 | 0.02±0.76 |
|  |  |
| ΔFEV1/FVC Ratio | -0.01±0.22 |
| ΔMethylated value of CpG sites at chr16:86528639 | 0.18±0.73 |
| ΔMethylated value of CpG sites at chr11:89900493 | 0.49±3.80 |
| ΔMethylated value of CpG sites at chr6:168708413 | -0.21±1.06 |
| ΔMethylated value of CpG sites at chr11:89900518 | 0.36±3.79 |
| ΔMethylated value of CpG sites at chr16:86528603 | 0.27±1.02 |
| ΔMethylated value of CpG sites at chr16:86528620 | 0.28±1.13 |
| ΔMethylated value of CpG sites at chr16:86528600 | 0.26±0.98 |
| ΔMethylated value of CpG sites at chr16:86528611 | 0.32±1.29 |
| ΔMethylated value of CpG sites at chr20:61992129 | 0.35±1.69 |
| ΔMethylated value of CpG sites at chr11:1103266 | 0.15±0.72 |
| ΔMethylated value of CpG sites at chr11:1103270 | 0.16±0.73 |
| ΔMethylated value of CpG sites at chr16:86528570 | 0.16±0.79 |

FEV1, Forced Expiratory Volume in one second; FVC, Forced Vital Capacity; SD, Standard Deviation.
