# Supplementary figures and images for: Genome-wide DNA methylation analysis of pulmonary function in middle and old-aged Chinese monozygotic twins
Source: Respir Res. 2021 Nov 22;22:300. doi: 10.1186/s12931-021-01896-5 (PMC8609861; doi:10.1186/s12931-021-01896-5)

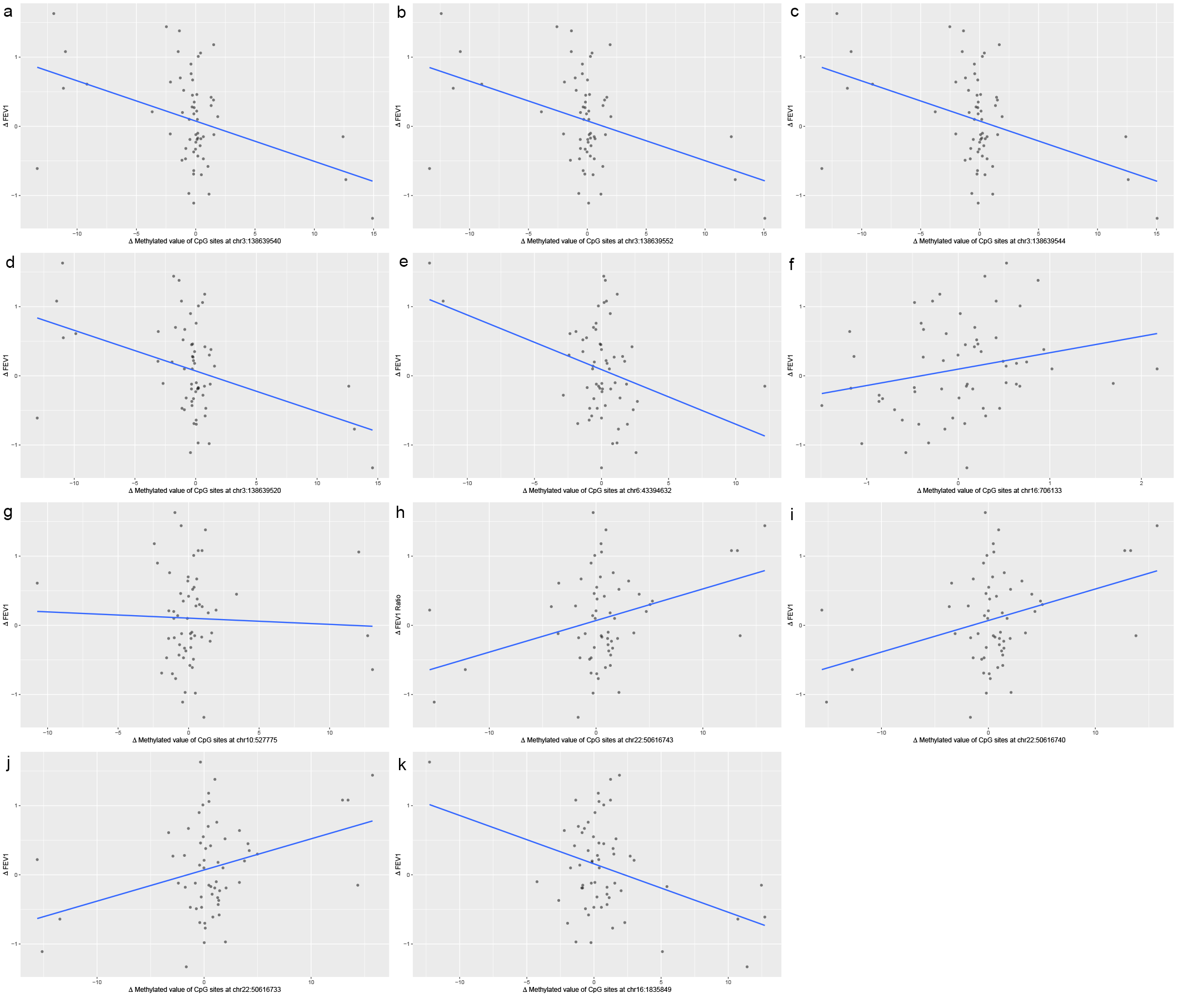

Supplement: Supplementary file 3 — Additional file 3: Figure S1. Scatter plots with regression lineshowing the association of Δ methylated value of CpG sites and Δ FEV1. The Δmethylation value of four CpG sites (f, h, i, j) were positively correlatedwith ΔFEV1, and the Δ methylation value of seven CpG sites (a,b,c,d,e,g,k) werenegatively correlated with ΔFEV1. [file 12931_2021_1896_MOESM3_ESM.tif]

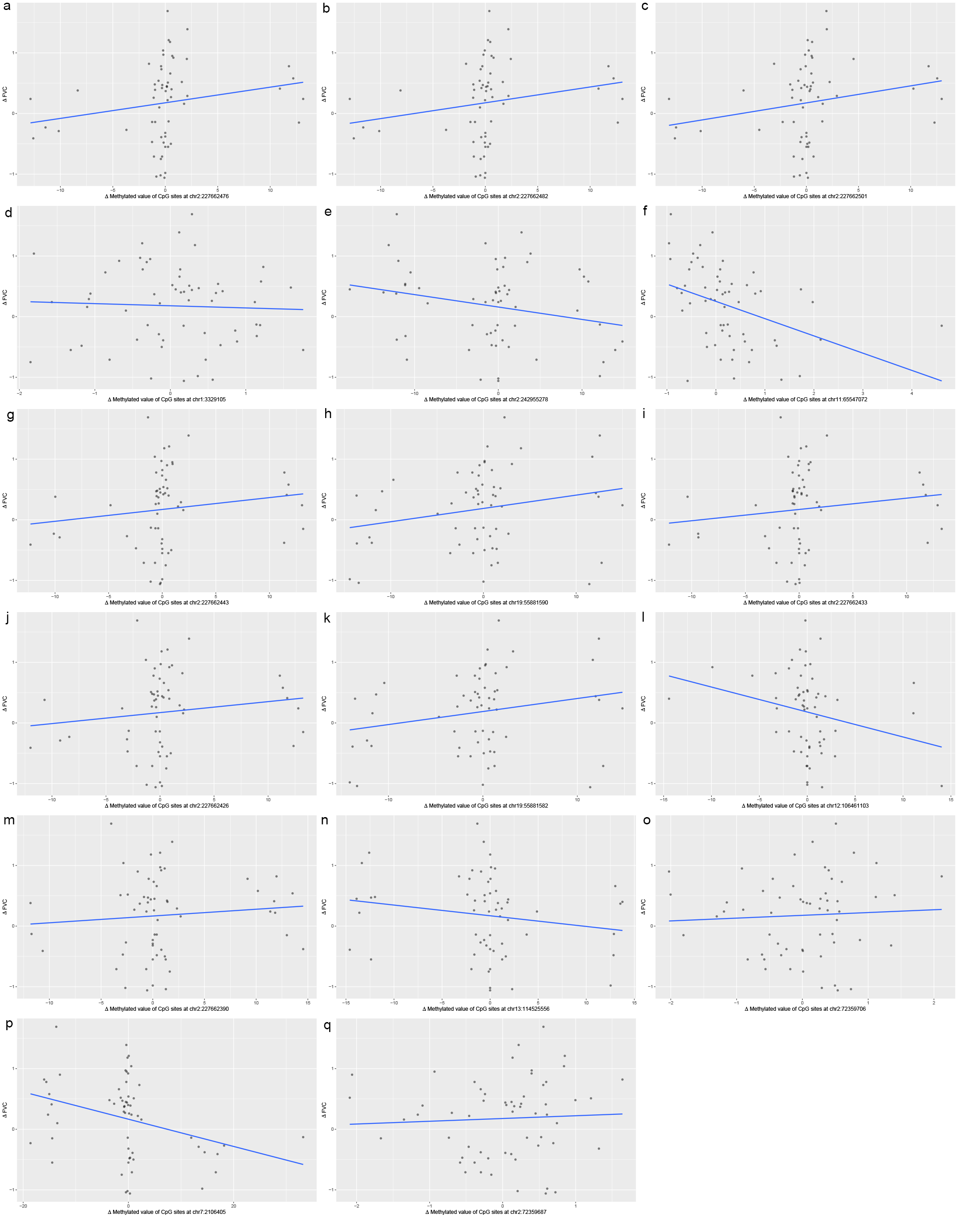

Supplement: Supplementary file 4 — Additional file 4: Figure S2. Scatter plot with regression line showing the association of Δ methylatedvalue of CpG sites and Δ FVC. The Δ methylation value of eleven CpG sites(a,b,c,g,h,i,j,k,m,o,q) were positively correlated with ΔFVC, and the Δmethylation value of six CpG sites (d,e,f,l,n,p) were negatively correlatedwith ΔFVC. [file 12931_2021_1896_MOESM4_ESM.tif]

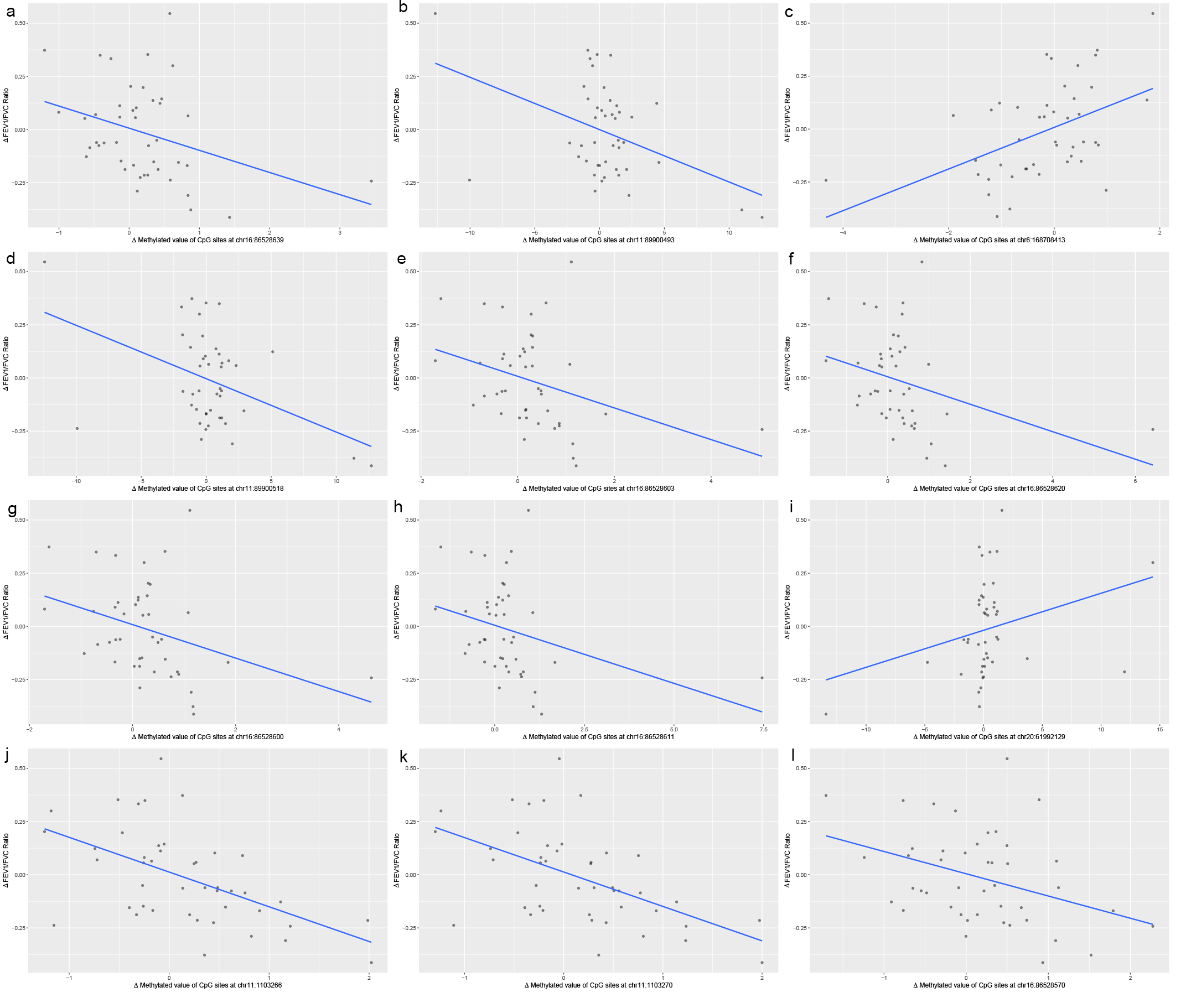

Supplement: Supplementary file 5 — Additional file 5: Figure S3. Scatter plots with regression line showing the association of Δ methylatedvalue of CpG sites and Δ FEV1/FVC. The Δ methylation value of two CpG sites(c,i) were positively correlated with ΔFEV1/FVC ratio, and the Δ methylation valueof ten CpG sites (a,b,d,e,f,g,h,j,k,l) were negatively correlated withΔFEV1/FVC ratio. [file 12931_2021_1896_MOESM5_ESM.tif]

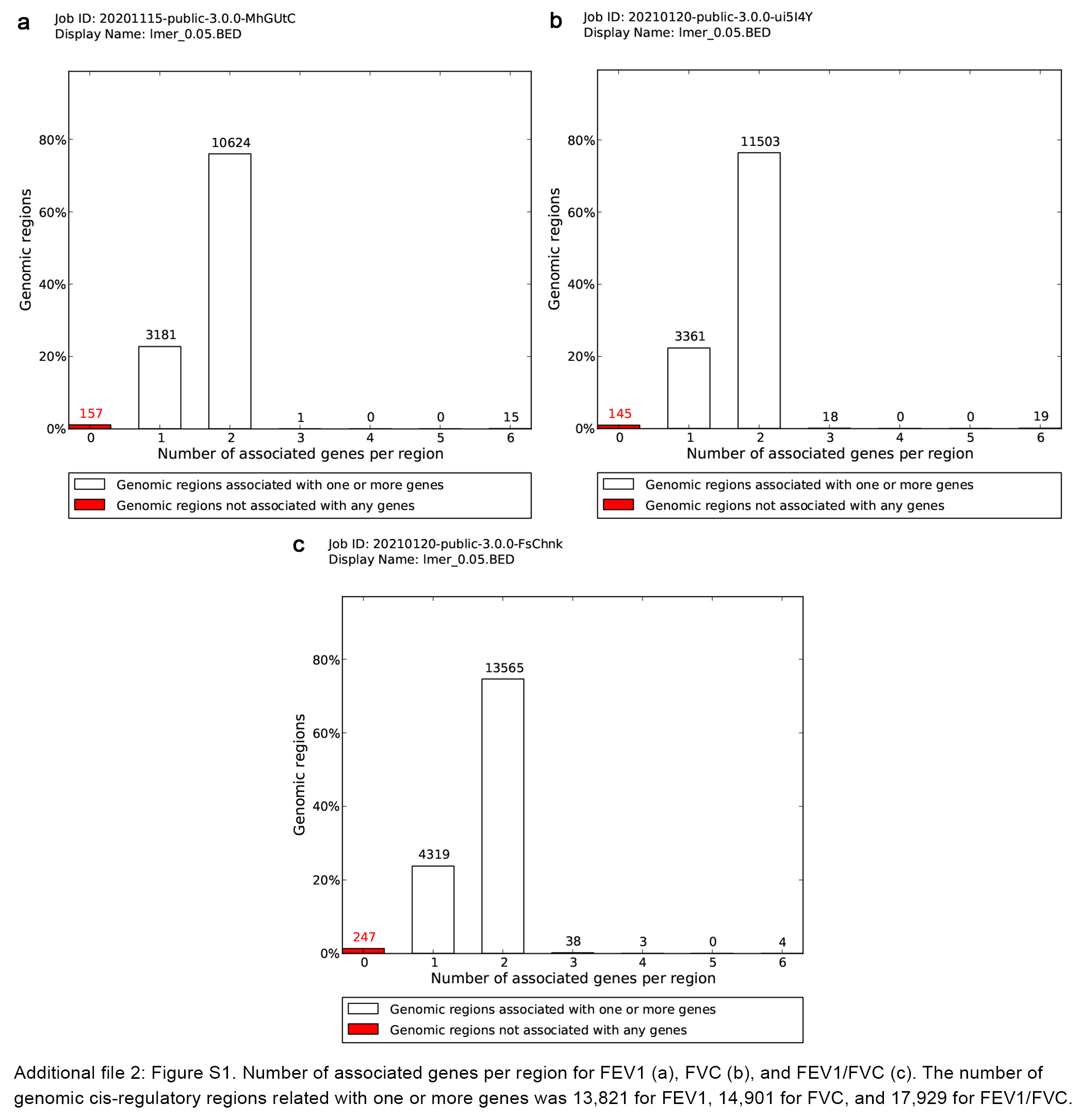

Supplement: Supplementary file 6 — Additional file 6: Figure S4. Number of associated genes per region for FEV1 (a), FVC (b), and FEV1/FVC (c). The number of genomiccis-regulatory regions related with one or more genes was 13,821 for FEV1,14,901 for FVC, and 17,929 for FEV1/FVC. [file 12931_2021_1896_MOESM6_ESM.tif]

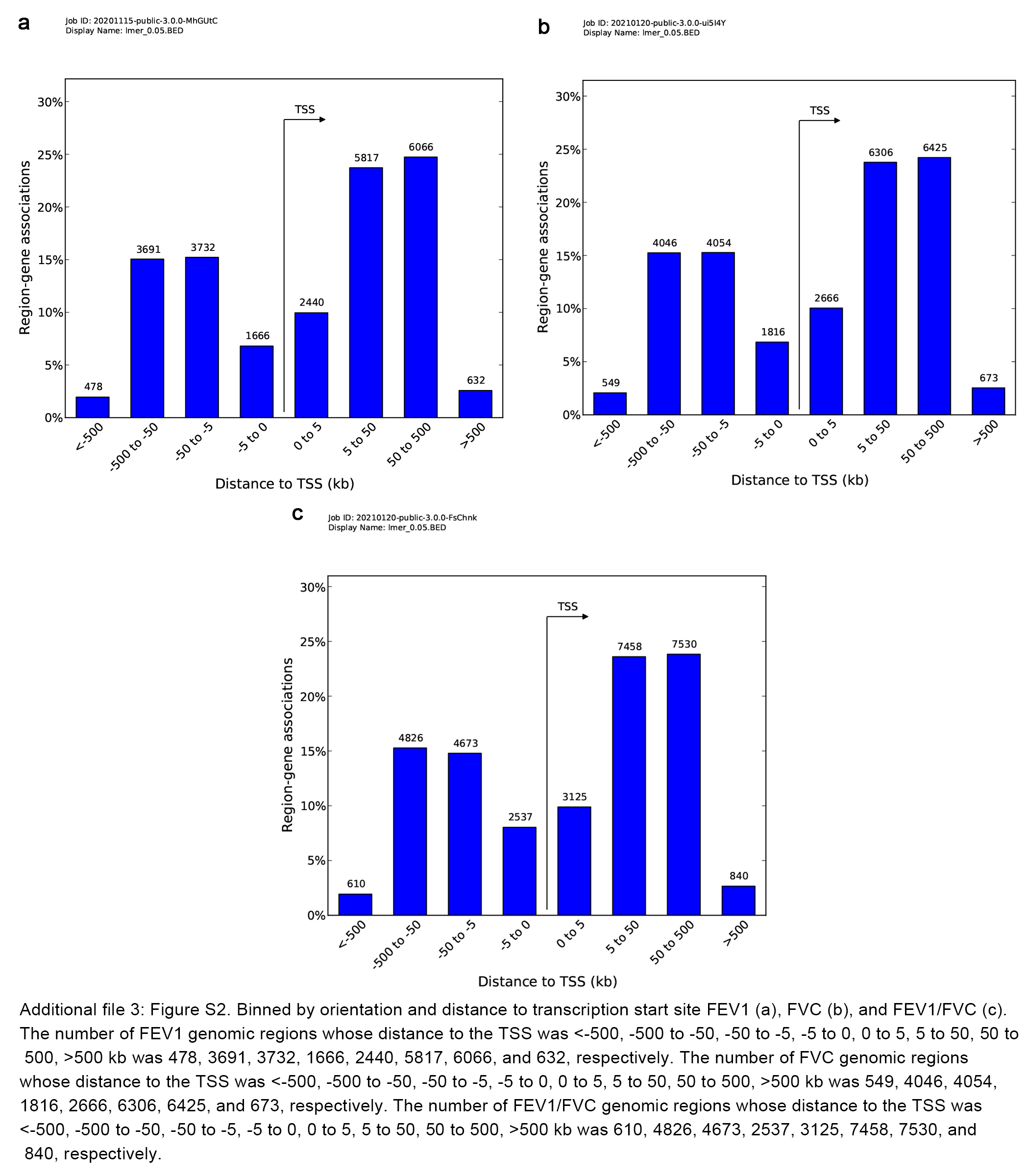

Supplement: Supplementary file 7 — Additional file 7: Figure S5. Binned by orientation and distance to transcription start site FEV1 (a),FVC (b), and FEV1/FVC (c). Thenumber of FEV1 genomic regions whose distance to the TSS was <-500, -500 to-50, -50 to -5, -5 to 0, 0 to 5, 5 to 50, 50 to 500, >500 kb was 478, 3691,3732, 1666, 2440, 5817, 6066, and 632, respectively. The number of FVC genomicregions whose distance to the TSS was <-500, -500 to -50, -50 to -5, -5 to0, 0 to 5, 5 to 50, 50 to 500, >500 kb was 549, 4046, 4054, 1816, 2666,6306, 6425, and 673, respectively. The number of FEV1/FVC genomic regions whosedistance to the TSS was <-500, -500 to -50, -50 to -5, -5 to 0, 0 to 5, 5 to50, 50 to 500, >500 kb was 610, 4826, 4673, 2537, 3125, 7458, 7530, and 840,respectively. [file 12931_2021_1896_MOESM7_ESM.tif]

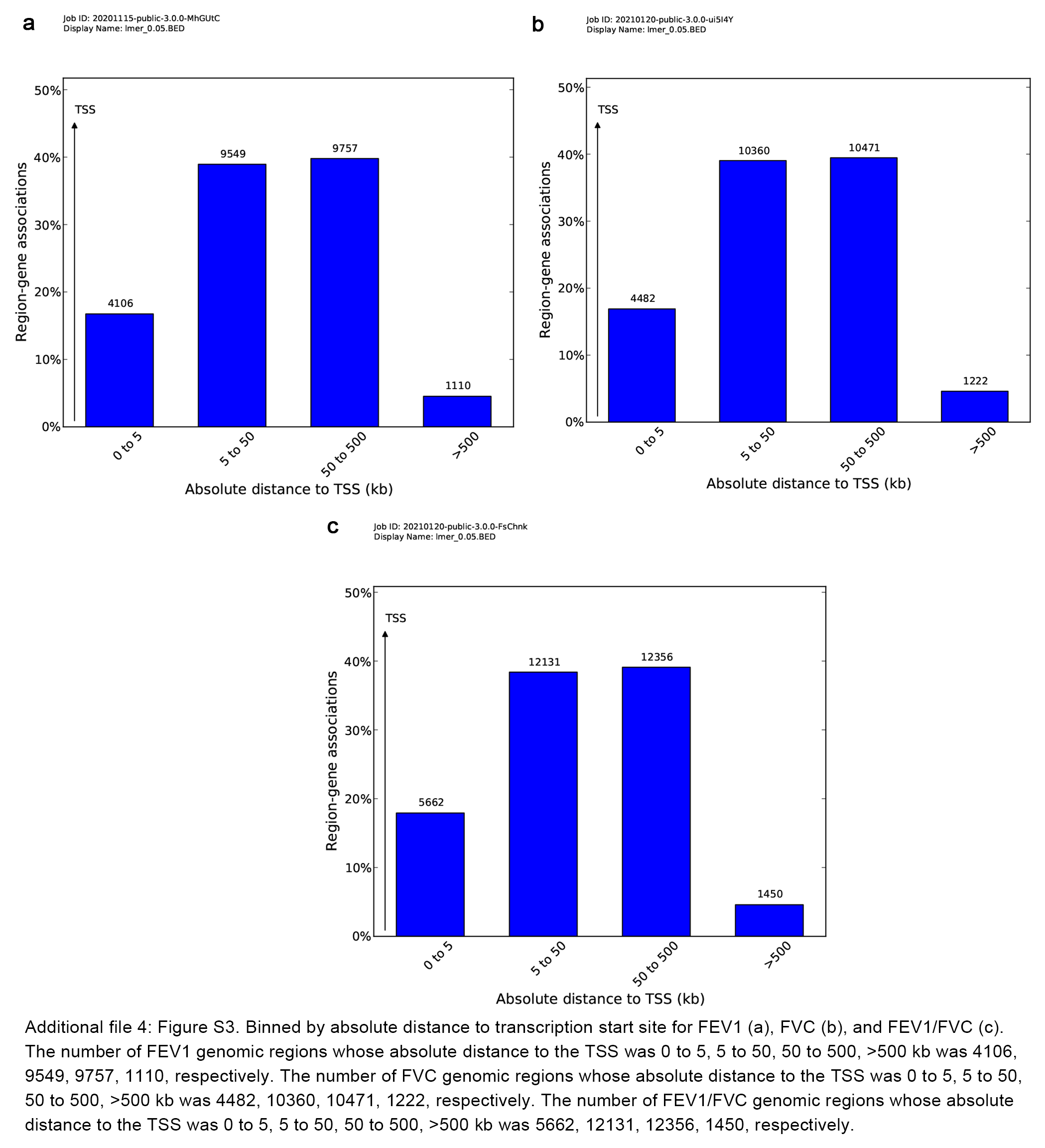

Supplement: Supplementary file 8 — Additional file 8: Figure S6. Binned by absolute distance to transcription start site for FEV1 (a), FVC(b), and FEV1/FVC (c). Thenumber of FEV1 genomic regions whose absolute distance to the TSS was 0 to 5, 5to 50, 50 to 500, >500 kb was 4106, 9549, 9757, 1110, respectively. Thenumber of FVC genomic regions whose absolute distance to the TSS was 0 to 5, 5to 50, 50 to 500, >500 kb was 4482, 10360, 10471, 1222, respectively. Thenumber of FEV1/FVC genomic regions whose absolute distance to the TSS was 0 to5, 5 to 50, 50 to 500, >500 kb was 5662, 12131, 12356, 1450, respectively. [file 12931_2021_1896_MOESM8_ESM.tif]

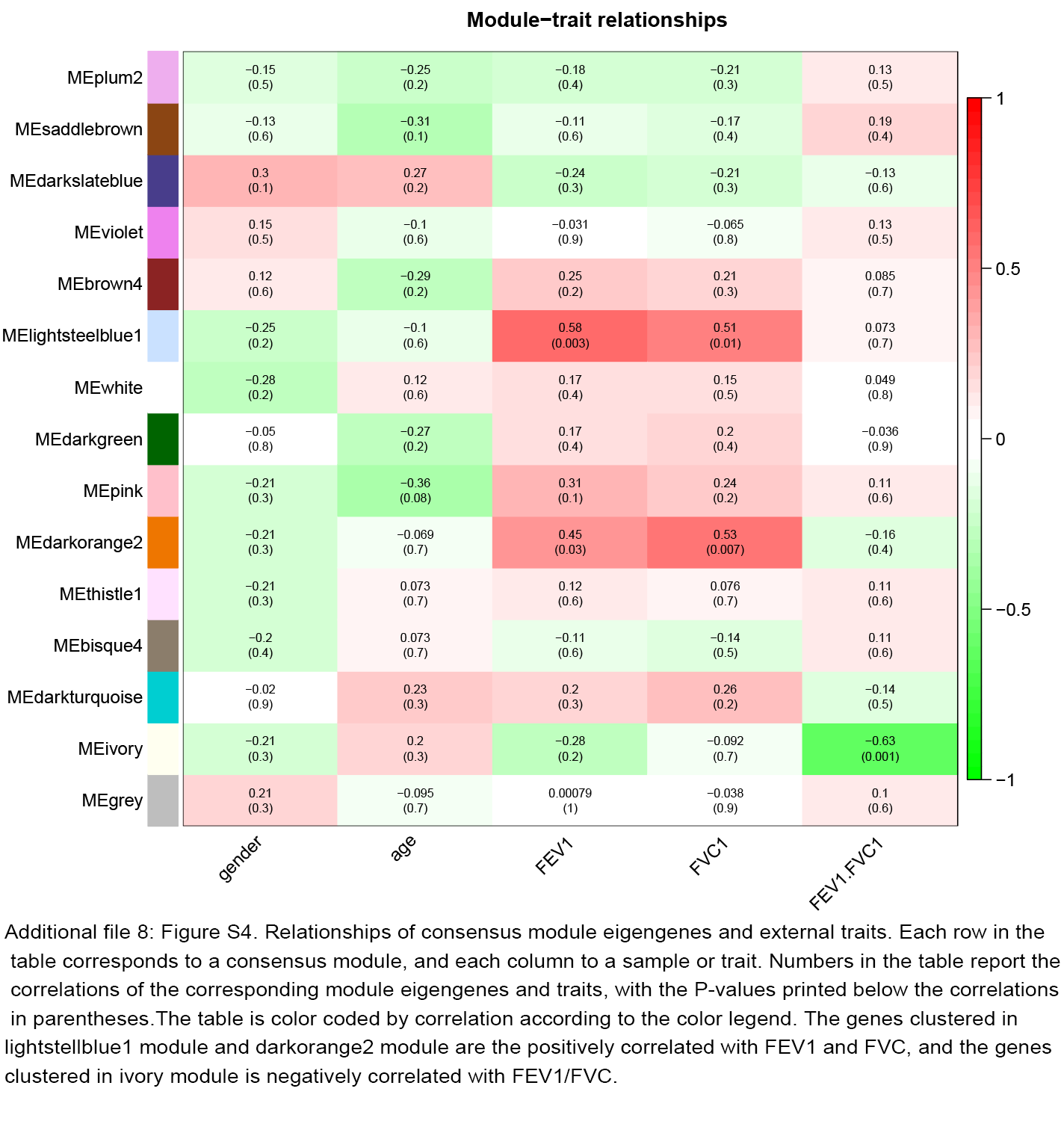

Supplement: Supplementary file 12 — Additional file 12: Figure S7. Relationships of consensus module eigengenes and external traits. Each row inthe table corresponds to a consensus module, and each column to a sample ortrait. Numbers in the table report the correlations of the corresponding moduleeigengenes and traits, with the P-valuesprinted below the correlations in parentheses. The table is color coded bycorrelation according to the color legend. The genes clustered inlightstellblue1 module and darkorange2 module are the positively correlatedwith FEV1 and FVC, and the genes clustered in ivory module is negativelycorrelated with FEV1/FVC. [file 12931_2021_1896_MOESM12_ESM.tif]
