# Supplementary material for: Genome-wide DNA methylation analysis of pulmonary function in middle and old-aged Chinese monozygotic twins
Source: Respir Res. 2021 Nov 22;22:300. doi: 10.1186/s12931-021-01896-5 (PMC8609861; doi:10.1186/s12931-021-01896-5)
Supplement: Supplementary file 9 — Additional file 9: Table S3. Significantfunctional clusters biological process related to FEV1 by GREAT using binomialtest. [file 12931_2021_1896_MOESM9_ESM.docx]

Table S3. Significant functional clusters biological process related to FEV1 by GREAT using binomial test.

| Ontology | Term Name | Binom Raw *P*-Value | Binom FDR Q-Value | Binom Fold Enrichment | Binom Expected | Binom Observed Region Hits |
| --- | --- | --- | --- | --- | --- | --- |
| GO Biological Process | negative regulation of phospholipid biosynthetic process | 2.30E-112 | 2.40E-108 | 22.67703 | 5.159407 | 117 |
| GO Biological Process | negative regulation of phosphatidylinositol biosynthetic process | 2.17E-88 | 5.67E-85 | 26.3148 | 3.268123 | 86 |
| GO Biological Process | platelet-derived growth factor receptor-beta signaling pathway | 3.33E-70 | 5.80E-67 | 12.38927 | 7.91007 | 98 |
| GO Biological Process | peptidyl-histidine dephosphorylation | 5.57E-63 | 8.31E-60 | 169.0452 | 0.2011297 | 34 |
| GO Biological Process | negative regulation of platelet activation | 2.52E-57 | 2.63E-54 | 9.44086 | 9.95672 | 94 |
| GO Biological Process | epithelial-mesenchymal cell signaling | 3.35E-57 | 3.18E-54 | 6.273671 | 20.24333 | 127 |
| GO Biological Process | response to vitamin K | 2.80E-54 | 2.44E-51 | 22.28008 | 2.513457 | 56 |
| GO Biological Process | negative regulation of ATP citrate synthase activity | 3.25E-44 | 1.06E-41 | 46.7241 | 0.7276759 | 34 |
| GO Biological Process | regulation of lipid biosynthetic process | 2.95E-37 | 5.31E-35 | 2.153961 | 159.7058 | 344 |
| GO Biological Process | regulation of Cdc42 GTPase activity | 1.13E-38 | 2.28E-36 | 5.044984 | 20.41632 | 103 |
| GO Molecular Function | platelet-derived growth factor binding | 1.75E-66 | 6.45E-63 | 6.609811 | 21.48322 | 142 |
| GO Molecular Function | potassium:chloride symporter activity | 2.13E-59 | 2.62E-56 | 27.73878 | 2.018834 | 56 |
| GO Molecular Function | G-protein coupled neurotensin receptor activity | 8.67E-45 | 7.99E-42 | 29.13609 | 1.40719 | 41 |
| GO Molecular Function | low voltage-gated calcium channel activity | 1.91E-44 | 1.41E-41 | 18.17954 | 2.750345 | 50 |
| GO Molecular Function | cAMP response element binding protein binding | 1.41E-38 | 5.76E-36 | 9.067368 | 7.058278 | 64 |
| GO Molecular Function | CTD phosphatase activity | 8.13E-37 | 2.73E-34 | 11.66783 | 4.456699 | 52 |
| GO Molecular Function | cation:chloride symporter activity | 2.37E-28 | 4.36E-26 | 6.66686 | 8.699747 | 58 |
| GO Molecular Function | phospholipase D activity | 1.14E-22 | 1.45E-20 | 9.223775 | 3.902957 | 36 |
| GO Molecular Function | GKAP/Homer scaffold activity | 3.61E-22 | 4.03E-20 | 6.973027 | 6.166618 | 43 |
| GO Molecular Function | mitogen-activated protein kinase p38 binding | 9.76E-16 | 5.81E-14 | 6.090298 | 5.418454 | 33 |
| GO Cellular Component | endoplasmic reticulum lumen | 3.03E-58 | 1.28E-55 | 2.235903 | 224.0705 | 501 |
| GO Cellular Component | platelet alpha granule lumen | 1.11E-36 | 1.41E-34 | 2.811148 | 72.21249 | 203 |
| GO Cellular Component | secretory granule lumen | 6.45E-34 | 7.42E-32 | 2.539451 | 87.81424 | 223 |
| GO Cellular Component | cytoplasmic membrane-bounded vesicle lumen | 6.81E-31 | 6.63E-29 | 2.35353 | 99.4251 | 234 |
| GO Cellular Component | apical cortex | 8.24E-31 | 7.44E-29 | 10.48218 | 4.3884 | 46 |
| GO Cellular Component | actomyosin | 1.67E-30 | 1.41E-28 | 2.528751 | 79.48588 | 201 |
| GO Cellular Component | platelet alpha granule | 4.25E-29 | 3.36E-27 | 2.437336 | 84.10822 | 205 |
| GO Cellular Component | AP-5 adaptor complex | 2.30E-26 | 1.71E-24 | 27.64928 | 0.8680153 | 24 |
| GO Cellular Component | SNARE complex | 2.73E-21 | 1.81E-19 | 3.032503 | 33.30582 | 101 |
| Human Phenotype | Anomalous pulmonary venous return | 1.02E-52 | 3.15E-49 | 9.824472 | 8.550077 | 84 |
| Human Phenotype | Abnormality of the pulmonary veins | 3.00E-52 | 6.14E-49 | 8.087219 | 11.74693 | 95 |
| Human Phenotype | Decreased serum estradiol | 2.18E-47 | 2.23E-44 | 21.93261 | 2.234116 | 49 |
| Human Phenotype | Atrioventricular canal defect with right ventricle aorta and pulmonary atresia | 4.52E-37 | 2.31E-34 | 129.1041 | 0.1626594 | 21 |
| Human Phenotype | Pulmonary artery atresia | 2.22E-29 | 4.88E-27 | 13.99717 | 2.643391 | 37 |
| MSigDB Pathway | Ceramide signaling pathway | 3.85E-33 | 1.69E-30 | 2.874993 | 61.21754 | 176 |
| MSigDB Pathway | Genes involved in Glucagon signaling in metabolic regulation | 8.13E-28 | 2.68E-25 | 3.041519 | 44.05693 | 134 |
| MSigDB Pathway | Notch signaling pathway | 3.13E-26 | 6.88E-24 | 2.816631 | 50.41484 | 142 |
| MSigDB Pathway | Genes involved in Prostacyclin signalling through prostacyclin receptor | 4.99E-24 | 8.23E-22 | 4.684612 | 14.30215 | 67 |
| MSigDB Pathway | Genes involved in Na+/Cl- dependent neurotransmitter transporters | 2.11E-22 | 2.79E-20 | 3.601859 | 23.32129 | 84 |
| MSigDB Pathway | Cytokines and Inflammatory Response | 4.84E-21 | 4.91E-19 | 2.847985 | 38.6238 | 110 |
| MSigDB Pathway | Genes involved in Thromboxane signalling through TP receptor | 2.65E-20 | 2.33E-18 | 3.698719 | 19.73656 | 73 |
| MSigDB Pathway | Genes involved in Aquaporin-mediated transport | 1.73E-19 | 1.42E-17 | 2.336085 | 62.06966 | 145 |
| MSigDB Pathway | amb2 Integrin signaling | 4.56E-18 | 3.17E-16 | 2.696418 | 37.82797 | 102 |
| MSigDB Pathway | FOXA2 and FOXA3 transcription factor networks | 1.54E-14 | 6.79E-13 | 2.153133 | 58.05495 | 125 |
| PANTHER Pathway | Transcription regulation by bZIP transcription factor | 2.38E-29 | 3.61E-27 | 3.509946 | 33.04894 | 116 |
| PANTHER Pathway | General transcription regulation | 8.66E-19 | 6.58E-17 | 3.394359 | 22.09548 | 75 |
| PANTHER Pathway | Interferon-gamma signaling pathway | 1.63E-15 | 6.21E-14 | 2.736413 | 30.69712 | 84 |
| PANTHER Pathway | Hedgehog signaling pathway | 4.56E-14 | 1.39E-12 | 2.553232 | 33.29114 | 85 |
| PANTHER Pathway | Pyruvate metabolism | 3.30E-12 | 7.17E-11 | 4.943528 | 6.06854 | 30 |
| PANTHER Pathway | Metabotropic glutamate receptor group II pathway | 6.84E-12 | 1.30E-10 | 2.005447 | 58.83975 | 118 |
| PANTHER Pathway | Ornithine degradation | 3.96E-11 | 6.69E-10 | 5.594897 | 4.289623 | 24 |
