# Supplementary material for: Genome-wide DNA methylation analysis of pulmonary function in middle and old-aged Chinese monozygotic twins
Source: Respir Res. 2021 Nov 22;22:300. doi: 10.1186/s12931-021-01896-5 (PMC8609861; doi:10.1186/s12931-021-01896-5)
Supplement: Supplementary file 10 — Additional file 10: Table S4. Significantfunctional clusters biological process related to FVC by GREAT using binomialtest [file 12931_2021_1896_MOESM10_ESM.docx]

Table S4 Significant functional clusters biological process related to FVC by GREAT using binomial test.

| Ontology | Term name | Binom Raw *P*-Value | Binom FDR Q-Value | Binom Fold Enrichment | Binom Expected Region Hits | Binom Observed Region Hits |
| --- | --- | --- | --- | --- | --- | --- |
| GO Biological Process | negative regulation of granulocyte differentiation | 1.29E-66 | 1.68E-63 | 9.154191 | 12.23483 | 112 |
| GO Biological Process | response to vitamin K | 9.28E-65 | 8.08E-62 | 24.02514 | 2.705499 | 65 |
| GO Biological Process | regulation of branching involved in salivary gland morphogenesis by epithelial-mesenchymal signaling | 6.96E-57 | 2.51E-54 | 22.09246 | 2.670594 | 59 |
| GO Biological Process | cellular response to gonadotropin stimulus | 4.36E-56 | 1.52E-53 | 5.064429 | 29.8158 | 151 |
| GO Biological Process | positive regulation of metanephric mesenchymal cell migration by platelet-derived growth factor receptor-beta signaling pathway | 8.98E-55 | 2.93E-52 | 17.08419 | 3.746155 | 64 |
| GO Biological Process | negative regulation of phospholipid biosynthetic process | 1.82E-54 | 5.77E-52 | 13.14459 | 5.553615 | 73 |
| GO Biological Process | positive regulation of Cdc42 GTPase activity | 9.35E-54 | 2.79E-51 | 8.693291 | 10.6979 | 93 |
| GO Biological Process | negative regulation of phosphatidylinositol biosynthetic process | 3.49E-50 | 9.11E-48 | 16.77172 | 3.517826 | 59 |
| GO Biological Process | epithelial-mesenchymal cell signaling | 1.61E-45 | 3.29E-43 | 5.323534 | 21.79004 | 116 |
| GO Cellular Component | actomyosin | 5.08E-38 | 5.84E-36 | 2.676514 | 85.55906 | 229 |
| GO Cellular Component | NELF complex | 6.85E-33 | 7.22E-31 | 19.86584 | 1.761818 | 35 |
| GO Cellular Component | stress fiber | 2.40E-29 | 2.34E-27 | 2.604297 | 70.26849 | 183 |
| GO Cellular Component | actin filament bundle | 1.12E-28 | 9.41E-27 | 2.56927 | 71.22644 | 183 |
| GO Cellular Component | AP-5 adaptor complex | 1.90E-21 | 1.51E-19 | 22.47584 | 0.9343367 | 21 |
| GO Cellular Component | troponin complex | 4.67E-20 | 3.48E-18 | 13.56715 | 1.842687 | 25 |
| GO Cellular Component | telomerase holoenzyme complex | 5.17E-18 | 3.27E-16 | 11.06153 | 2.260085 | 25 |
| GO Cellular Component | P granule | 6.63E-18 | 3.99E-16 | 4.250635 | 12.70398 | 54 |
| GO Cellular Component | laminin-11 complex | 2.40E-17 | 1.38E-15 | 10.3398 | 2.417841 | 25 |
| GO Cellular Component | platelet alpha granule lumen | 3.69E-16 | 1.87E-14 | 2.045544 | 77.72995 | 159 |
| GO Molecular Function | potassium:chloride symporter activity | 1.08E-73 | 1.99E-70 | 30.83174 | 2.173085 | 67 |
| GO Molecular Function | cation:chloride symporter activity | 9.73E-50 | 4.49E-47 | 8.970086 | 9.364458 | 84 |
| GO Molecular Function | platelet-derived growth factor binding | 3.14E-36 | 1.05E-33 | 4.583852 | 23.12466 | 106 |
| GO Molecular Function | low voltage-gated calcium channel activity | 1.08E-35 | 3.33E-33 | 14.86242 | 2.960487 | 44 |
| GO Molecular Function | CTD phosphatase activity | 3.14E-33 | 8.91E-31 | 10.42271 | 4.797216 | 50 |
| GO Molecular Function | neurotransmitter transporter activity | 5.53E-28 | 1.07E-25 | 3.61935 | 29.28703 | 106 |
| GO Molecular Function | G-protein coupled neurotensin receptor activity | 4.34E-27 | 7.61E-25 | 19.14562 | 1.514707 | 29 |
| GO Molecular Function | 3-galactosyl-N-acetylglucosaminide 4-alpha-L-fucosyltransferase activity | 1.06E-26 | 1.70E-24 | 46.92396 | 0.4262215 | 20 |
| GO Molecular Function | cAMP response element binding protein binding | 1.22E-19 | 1.05E-17 | 5.791325 | 7.597571 | 44 |
| GO Molecular Function | mitogen-activated protein kinase p38 binding | 3.38E-17 | 2.04E-15 | 6.172357 | 5.832456 | 36 |
| Human Phenotype | Mildly elevated creatine phosphokinase | 2.21E-47 | 3.40E-44 | 6.171462 | 17.17583 | 106 |
| Human Phenotype | Neutral hyperaminoaciduria | 1.18E-46 | 1.46E-43 | 60.74123 | 0.5432884 | 33 |
| Human Phenotype | Difficulty climbing stairs | 4.27E-46 | 4.38E-43 | 5.609275 | 19.96693 | 112 |
| Human Phenotype | Decreased serum estradiol | 2.81E-43 | 1.92E-40 | 19.54412 | 2.404816 | 47 |
| Human Phenotype | Elevated circulating parathyroid hormone (PTH) level | 2.83E-29 | 3.35E-27 | 7.456462 | 7.376152 | 55 |
| MSigDB Pathway | Genes involved in Elongation arrest and recovery | 2.20E-47 | 1.45E-44 | 6.790015 | 14.43296 | 98 |
| MSigDB Pathway | PKC-catalyzed phosphorylation of inhibitory phosphoprotein of myosin phosphatase | 8.48E-34 | 3.73E-31 | 3.062915 | 53.21727 | 163 |
| MSigDB Pathway | Notch signaling pathway | 4.40E-29 | 1.16E-26 | 2.856257 | 54.26683 | 155 |
| MSigDB Pathway | Genes involved in Prostacyclin signalling through prostacyclin receptor | 2.11E-28 | 4.63E-26 | 4.936696 | 15.39491 | 76 |
| MSigDB Pathway | Genes involved in Formation of RNA Pol II elongation complex | 1.11E-27 | 2.10E-25 | 3.756398 | 26.62125 | 100 |
| MSigDB Pathway | Genes involved in RNA Polymerase II Pre-transcription Events | 1.33E-27 | 2.19E-25 | 3.087523 | 42.10495 | 130 |
| MSigDB Pathway | Vibrio cholerae infection | 2.56E-23 | 3.76E-21 | 2.384867 | 70.8635 | 169 |
| MSigDB Pathway | Genes involved in Glucagon signaling in metabolic regulation | 7.67E-22 | 1.01E-19 | 2.678018 | 47.42313 | 127 |
| MSigDB Pathway | Ceramide signaling pathway | 1.07E-13 | 4.56E-12 | 2.033541 | 65.89492 | 134 |
| MSigDB Pathway | Genes involved in Gamma-carboxylation, transport, and amino-terminal cleavage of proteins | 7.63E-13 | 2.80E-11 | 4.626134 | 7.34955 | 34 |
| PANTHER Pathway | Transcription regulation by bZIP transcription factor | 1.57E-31 | 2.39E-29 | 3.513795 | 35.57407 | 125 |
| PANTHER Pathway | General transcription regulation | 1.03E-24 | 7.84E-23 | 3.742058 | 23.7837 | 89 |
| PANTHER Pathway | Pyruvate metabolism | 8.14E-13 | 3.09E-11 | 4.8988 | 6.532212 | 32 |
| PANTHER Pathway | Adrenaline and noradrenaline biosynthesis | 2.90E-11 | 8.82E-10 | 2.401932 | 30.80853 | 74 |
| PANTHER Pathway | Interferon-gamma signaling pathway | 2.54E-10 | 6.44E-09 | 2.2698 | 33.04255 | 75 |
