# Supplementary material for: Genome-wide DNA methylation analysis of pulmonary function in middle and old-aged Chinese monozygotic twins
Source: Respir Res. 2021 Nov 22;22:300. doi: 10.1186/s12931-021-01896-5 (PMC8609861; doi:10.1186/s12931-021-01896-5)
Supplement: Supplementary file 11 — Additional file 11: Table S5. Significantfunctional clusters biological process related to FEV1/FVC by GREAT usingbinomial test. [file 12931_2021_1896_MOESM11_ESM.docx]

Table S5. Significant functional clusters biological process related to FEV1/FVC by GREAT using binomial test.

| Ontology | Term name | Binom Raw *P*-Value | Binom FDR Q-Value | Binom Fold Enrichment | Binom Expected Region Hits | Binom Observed Region Hits |
| --- | --- | --- | --- | --- | --- | --- |
| GO Biological Process | negative regulation of phosphatidylinositol biosynthetic process | 6.17E-135 | 2.15E-131 | 29.64961 | 4.249635 | 126 |
| GO Biological Process | platelet-derived growth factor receptor-beta signaling pathway | 1.15E-111 | 2.00E-108 | 14.19447 | 10.28569 | 146 |
| GO Biological Process | negative regulation of interleukin-4 biosynthetic process | 2.92E-92 | 3.05E-89 | 72.43991 | 0.8696863 | 63 |
| GO Biological Process | negative regulation of mast cell differentiation | 2.92E-92 | 3.05E-89 | 72.43991 | 0.8696863 | 63 |
| GO Biological Process | mitral valve formation | 9.59E-91 | 8.34E-88 | 38.83544 | 1.956975 | 76 |
| GO Biological Process | negative regulation of platelet activation | 9.50E-86 | 7.63E-83 | 10.34988 | 12.94701 | 134 |
| GO Biological Process | transcriptional activation by promoter-enhancer looping | 1.36E-77 | 1.01E-74 | 41.96639 | 1.501201 | 63 |
| GO Biological Process | epithelial-mesenchymal cell signaling | 2.93E-64 | 1.18E-61 | 5.812409 | 26.32299 | 153 |
| GO Biological Process | activation of Cdc42 GTPase activity | 3.00E-58 | 8.25E-56 | 14.56942 | 5.079131 | 74 |
| GO Cellular Component | endoplasmic reticulum lumen | 3.65E-62 | 7.69E-60 | 2.107319 | 291.3654 | 614 |
| GO Cellular Component | transcriptional repressor complex | 2.81E-52 | 3.23E-50 | 2.458978 | 151.2823 | 372 |
| GO Cellular Component | BAT3 complex | 4.27E-33 | 3.60E-31 | 28.87024 | 1.039133 | 30 |
| GO Cellular Component | apical cortex | 1.00E-32 | 7.91E-31 | 9.287875 | 5.706364 | 53 |
| GO Cellular Component | platelet alpha granule lumen | 2.75E-32 | 2.05E-30 | 2.438764 | 93.90001 | 229 |
| GO Cellular Component | exon-exon junction complex | 6.03E-31 | 4.24E-29 | 6.623627 | 9.662379 | 64 |
| GO Cellular Component | fibrillar center | 7.31E-30 | 4.40E-28 | 31.53343 | 0.8245218 | 26 |
| GO Cellular Component | ribonucleoprotein granule | 8.94E-30 | 5.14E-28 | 2.216294 | 115.5082 | 256 |
| GO Cellular Component | cytoplasmic mRNA processing body | 3.58E-27 | 1.89E-25 | 2.54511 | 69.15221 | 176 |
| GO Cellular Component | SNARE complex | 5.43E-27 | 2.75E-25 | 3.024808 | 43.30853 | 131 |
| GO Molecular Function | platelet-derived growth factor binding | 3.83E-103 | 1.41E-99 | 7.338399 | 27.93525 | 205 |
| GO Molecular Function | phospholipid-hydroperoxide glutathione peroxidase activity | 3.90E-56 | 2.88E-53 | 78.34492 | 0.4722706 | 37 |
| GO Molecular Function | low voltage-gated calcium channel activity | 9.99E-55 | 6.14E-52 | 17.61571 | 3.576354 | 63 |
| GO Molecular Function | histone deacetylase inhibitor activity | 1.84E-44 | 5.21E-42 | 28.59754 | 1.43369 | 41 |
| GO Molecular Function | platelet-derived growth factor receptor binding | 4.57E-43 | 1.20E-40 | 4.391964 | 30.28258 | 133 |
| GO Molecular Function | potassium:chloride symporter activity | 7.68E-43 | 1.89E-40 | 18.28468 | 2.625149 | 48 |
| GO Molecular Function | diacylglycerol O-acyltransferase activity | 1.94E-39 | 4.47E-37 | 19.2202 | 2.23723 | 43 |
| GO Molecular Function | UDP-sugar diphosphatase activity | 1.02E-36 | 2.10E-34 | 58.68241 | 0.443063 | 26 |
| GO Molecular Function | cAMP response element binding protein binding | 9.55E-35 | 1.76E-32 | 7.299999 | 9.178084 | 67 |
| GO Molecular Function | mitogen-activated protein kinase p38 binding | 1.19E-21 | 7.41E-20 | 6.386807 | 7.045774 | 45 |
| Human Phenotype | Childhood onset short-limb short stature | 3.05E-51 | 2.68E-48 | 27.86533 | 1.722571 | 48 |
| Human Phenotype | Enlarged thorax | 3.72E-50 | 2.86E-47 | 3.228992 | 70.61027 | 228 |
| Human Phenotype | Reduced factor VII activity | 4.08E-43 | 1.93E-40 | 31.97771 | 1.188328 | 38 |
| Human Phenotype | Abnormality of alkaline phosphatase activity | 1.15E-39 | 4.41E-37 | 3.346229 | 50.80346 | 170 |
| Human Phenotype | Decreased serum estradiol | 5.03E-36 | 1.55E-33 | 15.14585 | 2.905086 | 44 |
| MSigDB Pathway | Ceramide signaling pathway | 5.35E-44 | 3.53E-41 | 2.914465 | 79.60295 | 232 |
| MSigDB Pathway | Genes involved in Elongation arrest and recovery | 2.31E-41 | 1.02E-38 | 5.678094 | 17.43543 | 99 |
| MSigDB Pathway | Mechanism of Gene Regulation by Peroxisome Proliferators via PPARa(alpha) | 2.74E-33 | 9.05E-31 | 2.19539 | 133.917 | 294 |
| MSigDB Pathway | Signaling events mediated by HDAC Class I | 3.70E-33 | 9.76E-31 | 2.522044 | 87.62735 | 221 |
| MSigDB Pathway | Notch signaling pathway | 4.98E-31 | 9.39E-29 | 2.730495 | 65.55589 | 179 |
| MSigDB Pathway | Genes involved in Formation of RNA Pol II elongation complex | 1.19E-28 | 1.97E-26 | 3.513765 | 32.15923 | 113 |
| MSigDB Pathway | RXR and RAR heterodimerization with other nuclear receptor | 8.28E-27 | 9.94E-25 | 2.995453 | 44.06679 | 132 |
| MSigDB Pathway | Genes involved in Glucagon-type ligand receptors | 2.90E-25 | 2.73E-23 | 3.025622 | 40.32229 | 122 |
| MSigDB Pathway | Genes involved in mRNA Splicing | 7.70E-25 | 6.77E-23 | 2.3724 | 77.13706 | 183 |
| MSigDB Pathway | FOXA2 and FOXA3 transcription factor networks | 1.08E-24 | 8.94E-23 | 2.384405 | 75.49054 | 180 |
| PANTHER Pathway | Transcription regulation by bZIP transcription factor | 2.74E-27 | 4.16E-25 | 3.04832 | 42.9745 | 131 |
| PANTHER Pathway | p53 pathway by glucose deprivation | 4.44E-26 | 3.38E-24 | 3.573314 | 27.98523 | 100 |
| PANTHER Pathway | General transcription regulation | 3.80E-16 | 1.44E-14 | 2.854021 | 28.7314 | 82 |
| PANTHER Pathway | Metabotropic glutamate receptor group II pathway | 1.93E-15 | 5.86E-14 | 2.025851 | 76.51104 | 155 |
| PANTHER Pathway | General transcription by RNA polymerase I | 9.23E-15 | 2.34E-13 | 4.214412 | 10.44036 | 44 |
