# Supplementary material for: Genome-wide DNA methylation analysis of pulmonary function in middle and old-aged Chinese monozygotic twins
Source: Respir Res. 2021 Nov 22;22:300. doi: 10.1186/s12931-021-01896-5 (PMC8609861; doi:10.1186/s12931-021-01896-5)
Supplement: Supplementary file 13 — Additional file 13: Table S6. The results ofenrichment analysis for genes clustered in lightsteelblue1 module by DAVID tool. [file 12931_2021_1896_MOESM13_ESM.docx]

Table S6. The results of enrichment analysis for genes clustered in lightsteelblue1 module by DAVID tool

|  | Category | Term | Count | P-Value |
| --- | --- | --- | --- | --- |
| *GO-function* | GO-BP | positive regulation of protein secretion | 9 | 1.80E-06 |
|  | GO-BP | positive regulation of cell division | 8 | 1.20E-04 |
|  | GO-BP | platelet degranulation | 9 | 3.50E-03 |
|  | GO-BP | phospholipid metabolic process | 6 | 9.60E-03 |
|  | GO-BP | transport | 17 | 1.10E-02 |
|  | GO-BP | G-protein coupled receptor signaling pathway | 34 | 1.10E-02 |
|  | GO-BP | peptidyl-tyrosine phosphorylation | 10 | 1.20E-02 |
|  | GO-BP | retina development in camera-type eye | 6 | 1.30E-02 |
|  | GO-BP | positive regulation of protein localization to nucleus | 4 | 1.30E-02 |
|  | GO-BP | response to drug | 15 | 1.60E-02 |
|  | GO-BP | arachidonic acid secretion | 4 | 1.90E-02 |
|  | GO-BP | nervous system development | 14 | 2.20E-02 |
|  | GO-BP | negative regulation of endopeptidase activity | 8 | 2.70E-02 |
|  | GO-BP | cell wall macromolecule catabolic process | 3 | 2.80E-02 |
|  | GO-BP | metaphase plate congression | 3 | 3.30E-02 |
|  | GO-BP | hyaluronan metabolic process | 3 | 3.30E-02 |
|  | GO-BP | signal transduction | 39 | 3.50E-02 |
|  | GO-BP | circadian rhythm | 6 | 3.50E-02 |
|  | GO-BP | eye development | 4 | 3.50E-02 |
|  | GO-BP | axon guidance | 9 | 3.90E-02 |
|  | GO-BP | xenobiotic metabolic process | 6 | 4.00E-02 |
|  | GO-BP | negative regulation of neuron apoptotic process | 8 | 4.10E-02 |
|  | GO-BP | blood vessel remodeling | 4 | 4.10E-02 |
|  | GO-BP | semaphorin-plexin signaling pathway | 4 | 4.50E-02 |
|  | GO-BP | cell growth | 5 | 4.60E-02 |
|  | GO-BP | renal artery morphogenesis | 2 | 4.80E-02 |
|  | GO-BP | uterine wall breakdown | 2 | 4.80E-02 |
|  | GO-BP | potassium ion transport | 6 | 4.80E-02 |
|  | GO-BP | response to copper ion | 3 | 5.00E-02 |
|  | GO-CC | motile cilium | 8 | 2.50E-03 |
|  | GO-CC | plasma membrane | 123 | 9.30E-03 |
|  | GO-CC | extracellular region | 55 | 1.00E-02 |
|  | GO-CC | platelet alpha granule lumen | 6 | 1.10E-02 |
|  | GO-CC | integral component of plasma membrane | 48 | 1.90E-02 |
|  | GO-CC | integral component of membrane | 147 | 2.00E-02 |
|  | GO-CC | postsynaptic membrane | 11 | 3.50E-02 |
|  | GO-CC | Golgi cisterna membrane | 6 | 3.80E-02 |
|  | GO-CC | extracellular space | 44 | 4.30E-02 |
|  | GO-CC | neuronal cell body | 14 | 4.60E-02 |
|  | GO-CC | sarcoplasmic reticulum | 4 | 5.00E-02 |
|  | GO-MF | growth factor activity | 13 | 7.20E-04 |
|  | GO-MF | calcium ion binding | 32 | 1.90E-03 |
|  | GO-MF | phospholipase A2 activity | 5 | 6.90E-03 |
|  | GO-MF | serine-type endopeptidase inhibitor activity | 8 | 1.00E-02 |
|  | GO-MF | acetylgalactosaminyltransferase activity | 3 | 1.60E-02 |
|  | GO-MF | neuropeptide Y receptor activity | 3 | 2.00E-02 |
|  | GO-MF | calcium-dependent phospholipase A2 activity | 3 | 2.00E-02 |
|  | GO-MF | scavenger receptor activity | 5 | 3.10E-02 |
|  | GO-MF | lysozyme activity | 3 | 3.40E-02 |
|  | GO-MF | alpha-actinin binding | 3 | 4.00E-02 |
|  | GO-MF | protein tyrosine kinase activity | 8 | 4.80E-02 |
|  | GO-MF | N-acetyl-beta-glucosaminyl-glycoprotein 4-beta-N-acetylgalactosaminyltransferase activity | 2 | 4.90E-02 |
|  | GO-MF | G-protein coupled receptor activity | 26 | 4.90E-02 |
|  | GO-MF | copper ion binding | 5 | 5.00E-02 |
| *Pathway* | KEGG | Linoleic acid metabolism | 5 | 4.30E-03 |
|  | KEGG | Axon guidance | 9 | 9.60E-03 |
|  | KEGG | Cytokine-cytokine receptor interaction | 13 | 1.10E-02 |
|  | KEGG | Fat digestion and absorption | 5 | 1.30E-02 |
|  | KEGG | Arachidonic acid metabolism | 6 | 1.40E-02 |
|  | KEGG | alpha-Linolenic acid metabolism | 4 | 2.00E-02 |
|  | KEGG | Ether lipid metabolism | 5 | 2.00E-02 |
|  | KEGG | Metabolic pathways | 40 | 2.20E-02 |
|  | KEGG | Malaria | 5 | 2.70E-02 |
|  | KEGG | Olfactory transduction | 16 | 4.60E-02 |
