# Supplementary material for: Genome-wide DNA methylation analysis of pulmonary function in middle and old-aged Chinese monozygotic twins
Source: Respir Res. 2021 Nov 22;22:300. doi: 10.1186/s12931-021-01896-5 (PMC8609861; doi:10.1186/s12931-021-01896-5)
Supplement: Supplementary file 14 — Additional file 14: Table S7. The results ofenrichment analysis for genes clustered in darkorange2 module by DAVID tool [file 12931_2021_1896_MOESM14_ESM.docx]

Table S7. The results of enrichment analysis for genes clustered in darkorange2 module by DAVID tool

|  | Category | Term | Count | P-Value |
| --- | --- | --- | --- | --- |
| *GO-function* | GO-BP | negative regulation of exocytosis | 2 | 1.10E-02 |
|  | GO-BP | cell adhesion | 5 | 3.90E-02 |
|  | GO-CC | extracellular region | 12 | 4.30E-03 |
| *Pathway* | KEGG | Cell adhesion molecules (CAMs) | 3 | 4.70E-02 |
