# Supplementary material for: Genome-wide DNA methylation analysis of pulmonary function in middle and old-aged Chinese monozygotic twins
Source: Respir Res. 2021 Nov 22;22:300. doi: 10.1186/s12931-021-01896-5 (PMC8609861; doi:10.1186/s12931-021-01896-5)
Supplement: Supplementary file 15 — Additional file 15: Table S8. The results ofenrichment analysis for genes clustered in ivory module by DAVID tool. [file 12931_2021_1896_MOESM15_ESM.docx]

Table S8. The results of enrichment analysis for genes clustered in ivory module by DAVID tool

|  | Category | Term | Count | P-Value |
| --- | --- | --- | --- | --- |
| *GO-function* | GO-BP | plasminogen activation | 2 | 2.60E-02 |
|  | GO-BP | cell-cell signaling | 4 | 4.00E-02 |
|  | GO-BP | positive regulation of cell proliferation | 5 | 5.00E-02 |
|  | GO-CC | extracellular region | 14 | 1.40E-03 |
|  | GO-CC | intermediate filament | 4 | 5.60E-03 |
|  | GO-CC | cell surface | 7 | 7.40E-03 |
|  | GO-CC | extracellular space | 10 | 2.60E-02 |
|  | GO-MF | cytokine activity | 6 | 2.20E-04 |
